# Shared stream–lake patterns in diversity, rRNA-based activity and community assembly of bacteria and microeukaryotes under distinct hydrological regimes

**DOI:** 10.1093/femsec/fiag010

**Published:** 2026-02-11

**Authors:** Sofia Papadopoulou, Eva S Lindström

**Affiliations:** Department of Ecology and Genetics/Limnology, Uppsala University, Norbyvägen 18 D, 752 36 Uppsala, Sweden; Department of Ecology and Genetics/Limnology, Uppsala University, Norbyvägen 18 D, 752 36 Uppsala, Sweden

**Keywords:** aquatic microbial communities, RNA, DNA ratio, phantom taxa, community assembly, cytometric diversity, biogeography

## Abstract

Freshwater systems are shaped by hydrological connectivity, yet distinct microbial communities persist between lotic and lentic habitats. While bacterial biogeography across aquatic habitats has been widely explored, less is known about the spatiotemporal links of microeukaryotes to bacterial communities. Here, we investigated microbial diversity, rRNA-based activity and community assembly within a stream–lake network in Sweden under contrasting hydrological regimes. Using amplicon sequencing of both rRNA genes and transcripts, we found parallel patterns in bacterial and microeukaryotic alpha and beta diversity, with lower richness in lakes than in inlet streams. Bacterial phenotypic diversity, assessed by flow cytometry, captured biogeographic trends comparable to sequencing-based methods. Bacteria and microeukaryotes also appeared to be structured by similar assembly mechanisms, with environmental selection having a higher relative importance in lakes compared to streams. During low-flow periods, the catchment outlet became increasingly distinct from upstream communities, demonstrating dispersal limitation from lakes. Finally, phantom taxa, undetected in rRNA genes, were predominantly rare and exhibited disproportionately high RNA: DNA ratios compared to active taxa, underscoring the need for their careful handling. Our findings revealed habitat-driven microbial dynamics, despite pronounced seasonal shifts in hydrology.

## Teaser

Combined RNA- and DNA-based 16S and 18S rRNA amplicon sequencing revealed covarying bacterial and microeukaryotic diversity and community assembly dynamics across an aquatic network in central Sweden.

## Introduction

In hydrologically connected landscapes, microbial dispersal is predominantly unidirectional; cells are transported to downstream lakes via streams, rivers, subsurface flow, and surrounding soils (Lindström et al. 2006, Comte et al. 2017, Graham et al. 2017, Hermans et al. 2020). As these microbial communities migrate towards a catchment outlet, they are modified and reassembled (Stadler and del Giorgio 2021). Consequently, the lake community is not merely a sum of upstream contributions; rather, it is structured by the reshuffling of local communities and the varying incorporation of allochthonous taxa (Crevecoeur et al. 2022). Terrestrial habitats serve as a major microbial source for downstream communities, yet bacterial richness typically declines from soils and headwater streams to larger rivers and lake pelagic zones (Crump et al. 2012, Besemer et al. 2013, Ruiz-González et al. 2015, Sieber et al. 2020).

A potential driver of this spatial pattern lies in the typically longer water residence time (WRT) of lakes compared to upslope streams, enabling microbial communities to undergo species sorting in response to local conditions (Nelson et al. 2009, Logue and Lindström 2010, Crump et al. 2012, Adams et al. 2014). As water flows downstream, terrestrial and stream-derived microbes are progressively filtered out, while lake-adapted taxa become increasingly dominant (Crump et al. 2012, Read et al. 2015, Ruiz-González et al. 2015, Savio et al. 2015, Hassell et al. 2018). Priority effects may also contribute (Fukami 2015), with established lake taxa outcompeting later arrivals (Adams et al. 2014, Ruiz-González et al. 2015). As a result, microbial communities in lakes and large rivers are generally considered to be shaped primarily by local environmental selection (Logares et al. 2013, Souffreau et al. 2015, Wisnoski and Lennon 2021).

In contrast, mass effects tend to dominate in systems with shorter WRT, where rapid hydrological transport allows high microbial influxes to override local selection pressures (Crump et al. 2007, Nelson et al. 2009, Langenheder and Lindström 2019, Bambakidis et al. 2024). High-flow events, such as storms and floods, can mobilize distinct microbial assemblages from soils, increasing richness and altering community composition (Adams et al. 2014, Graupner et al. 2017, Caillon et al. 2021). Therefore, in highly connected freshwater networks, both legacy effects (Vass and Langenheder 2017, Crevecoeur et al. 2022) and contemporary ecological processes, shape biogeographic patterns (Hanson et al. 2012). Seasonal shifts in flow regimes are also expected to alter the relative importance of dispersal sources and community assembly processes (Comte et al. 2017), underscoring the need to integrate hydrology into microbial biogeographic frameworks (Lindström and Bergström 2004, de Melo et al. 2019).

An additional critical aspect is microbial reactivity following a dispersal event since the mere presence of a microbial taxon downstream does not necessarily indicate its successful establishment (Hanson et al. 2012, Sieber et al. 2020). To distinguish active from inactive bacterial taxa, comparisons of 16S rRNA genes (“DNA”) and transcripts (“RNA”) are often used, assessing their environmental responsiveness as the RNA: DNA ratio. Interpreting these ratios can be challenging, particularly in complex communities with diverse growth strategies (Blazewicz et al. 2013, Steven et al. 2017), as ribosome content is not always indicative of metabolic activity (Malmstrom et al. 2004, Sukenik et al. 2012). Additional complications include extracellular DNA (Dlott et al. 2015, Carini et al. 2016) and the occurrence of phantom taxa, i.e. taxa present in rRNA transcripts but undetected in rRNA genes (Klein et al. 2016). Despite these limitations, RNA: DNA ratios remain a useful proxy for microbial activity and can still be meaningfully applied when comparing samples with substantial differences in community structure (Steven et al. 2017). However, few studies have applied RNA: DNA ratios from a biogeographic perspective, especially for the 18S rRNA gene (Hu et al. 2016).

Here, we investigated patterns of microbial diversity, rRNA-based activity and community assembly processes across aquatic habitats within a stream–lake network in central Sweden. To our knowledge, we combined for the first time DNA and RNA data from both 16S and 18S rRNA amplicon sequencing to detect active and inactive microbial populations of bacteria and microeukaryotes across hydrologically distinct periods. We first hypothesized that (1a) taxonomic diversity, based on both rRNA genes and transcripts, was lower in lake pelagic environments than in the lotic habitats of the study system. We further hypothesized that (1b) phenotypic diversity of bacteria, estimated using flow cytometric fingerprints, was also lower in pelagic environments relative to streams. Our second hypothesis addressed rRNA-based activity, assessing whether (2) RNA: DNA ratios consistently peaked within a specific habitat type along transects from inflowing streams, through pelagic zones, to lake outlets. Finally, we hypothesized that (3) during low-flow periods, microbial communities at the catchment outlet became increasingly distinct from upstream communities. In particular, we were interested in identifying which fractions of the outlet community could be traced back to upstream habitats, and to what extent limited dispersal from upstream pelagic zones contributed to the observed patterns.

## Materials and methods

### Study system and sampling

**Figure 1:**
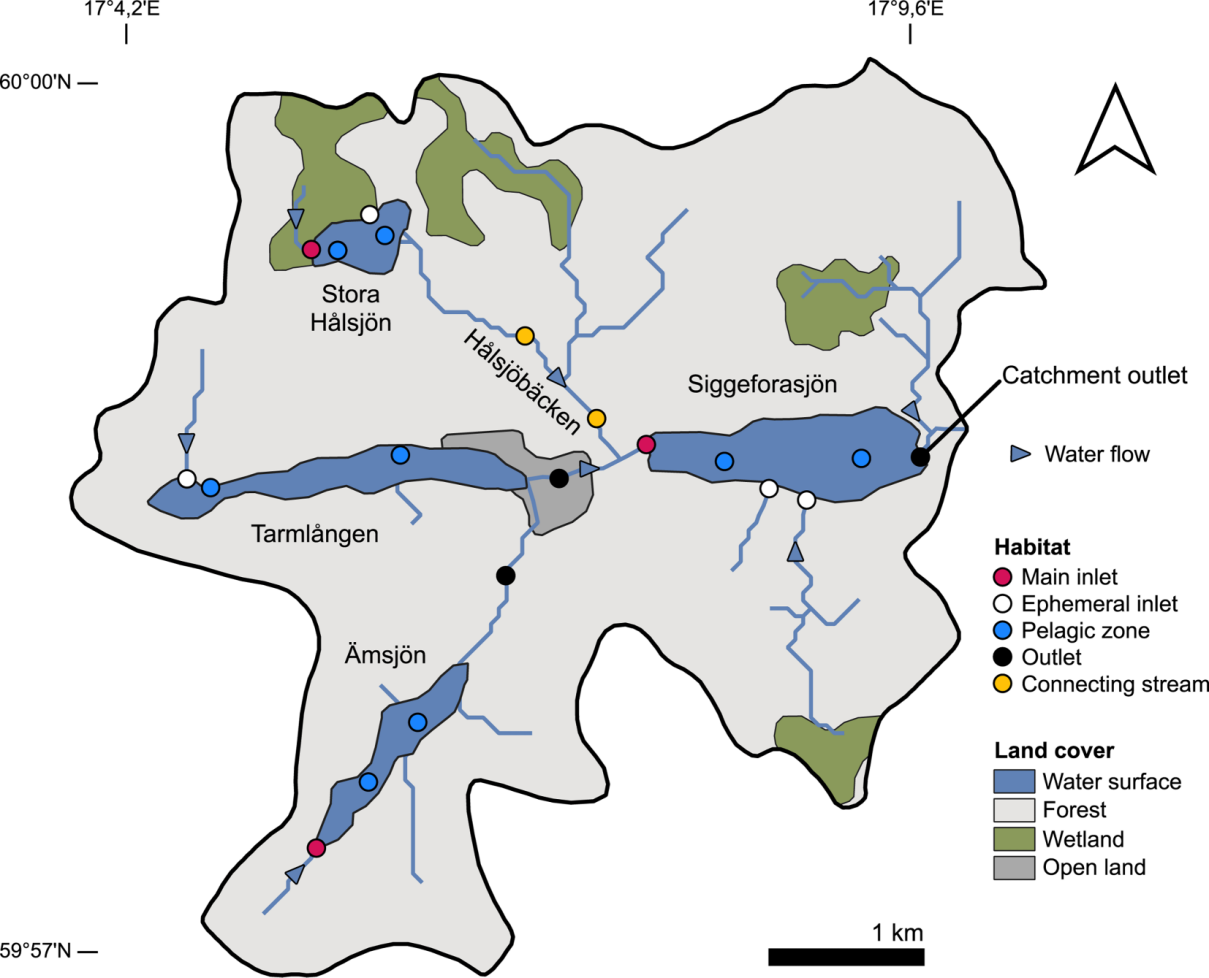
Map of the study system. Arrows indicate the water flow direction from the upstream lakes (Stora Hålsjön, Tarmlången and Ämsjön) to the downstream lake (Siggeforasjön) and the catchment outlet. Sampling locations are color-coded by habitat type; catchment land cover is also shown using distinct colors.

Our study focused on the catchment area of lake Siggeforasjön, located ∼30 km northwest of Uppsala, Sweden (Fig. 1). The system comprises three upstream lakes (Stora Hålsjön, Tarmlången, and Ämsjön), which drain into a single downstream lake (Siggeforasjön) via connecting streams. Three sampling campaigns were conducted in early summer (13 June), late summer (25 August), and autumn (2–3 November) of 2022 to capture seasonal variation in hydrological conditions. Samples were collected from lake inlets, pelagic zones, and outlet streams (sampling sites listed in Supplementary Table S1). Some inlet streams were ephemeral (i.e. temporary streams that appeared only after rainfall or snowmelt) and they were not sampled in early or late summer as they were dry. Additionally, the stream connecting lake Stora Hålsjön to the downstream lake (Hålsjöbäcken) was sampled, as it was expected to exhibit intermediate characteristics between inlet and outlet streams (hereafter called “stream”). Within each lake, two pelagic sampling sites were selected: one near the primary inflow and the other closer to the outflow. Water samples were collected at depths of 0.5–1.5 m using a Ruttner sampler, either from a boat (Stora Hålsjön, Ämsjön, and Siggeforasjön) or from docks (Tarmlången). In streams, surface water was collected by submerging a bottle against the flow, without disturbing the sediment. Samples intended for molecular or flow cytometric analyses (see below) were pre-filtered *in situ* using a 150 μm mesh to remove larger zooplankton.

### Physicochemical measurements

Water temperature and dissolved oxygen concentrations were measured *in situ* at each sampling location using a portable probe (HQ40d multi-parameter meter, HACH). Temperature and oxygen vertical profiles were taken in three lakes (Stora Hålsjön, Ämsjön and Siggeforasjön) to assess their mixing status. Profiles were not taken in lake Tarmlången due to the shallow depth at the sampling sites (0.5–1 m). Model-estimated daily data on total water flow and watercourse temperature at the catchment outlet for 2022 were obtained from the S-HYPE model through the Vattenwebb service of the Swedish Meteorological and Hydrological Institute, for the subcatchment SUBID: 8110 (Siggeforasjön). In addition, unfiltered water samples were collected at each location for the analysis of total organic carbon (TOC), total nitrogen (TN), and total phosphorus (TP) concentrations (see supplementary material for more details).

All statistical analyses were conducted in R (v4.4.2) and Rstudio (v2024.9.1) unless stated otherwise. To assess whether water temperature and dissolved oxygen concentrations varied significantly across sampling months (June, August and November), Kruskal–Wallis tests were applied using the *kruskal_test* function (rstatix v0.7.2). A non-parametric test was chosen in general when the assumptions of normality and homogeneity of variances were not met. Where significant differences were found, Dunn’s *post hoc* pairwise comparisons with Bonferroni correction were performed using the *dunn_test* function (rstatix). To evaluate the effects of habitat type (inlet, connecting stream, pelagic, outlet) and month on TOC, TN, and TP concentrations, two-way ANOVAs with Type III sums of squares were performed using the *Anova* function (car v3.1–3). When significant effects were detected, *post hoc* comparisons were carried out using Tukey’s honestly significant difference (HSD) test (*TukeyHSD*, stats v4.4.2).

### Bacterial abundance

Upon return to the laboratory, samples were passed through a 40 μm cell strainer (pluriSelect) and fixed with formalin to a final concentration of 1.76% formaldehyde. For each sample, a negative control (blank) was prepared by filtering the sample through a 0.2 μm filter. Aliquots of both samples and blanks were stored at −70°C until analysis. All cytometric measurements were performed using a CytoFLEX flow cytometer (Beckman Coulter) equipped with a 488 nm (blue) laser for excitation. Fluorescence emissions were detected using 525/40 (FL1) and 690/50 (FL3) filters. Side scatter was detected with the blue laser.

Prior to analysis, samples and blanks were diluted in phosphate-buffered saline. Particles were stained with SYBR Green I nucleic acid stain (Thermo Fisher Scientific) and incubated in the dark at room temperature, for exactly 15 min. Blanks were used to identify and exclude background noise. Flow cytometer settings included a flow rate of 30 μl min^−1^ and an acquisition time of 140 s per sample or until 100 000 events were recorded. Bacterial cell populations were identified and gated based on side scatter (SSC-A) and green fluorescence (FL1-A; EX 488, EM 525/40). Cyanobacterial populations were identified within the bacterial population based on green fluorescence from SYBR Green I staining (FL1-H; EX 488, EM 525/40) and the autofluorescence of chlorophyll-*a* (FL3-H; EX 488, EM 690/50). A single gate was applied uniformly across all samples to differentiate bacterial or cyanobacterial cells from background signals, and (cyano) bacterial particle concentrations were calculated per volume.

To assess the precision of flow cytometric measurements, the percentage of the coefficient of variation (CV%) was calculated based on the Poisson distribution as 100/√n, where n represented the total count of particles in the population of interest (i.e. bacteria; Shapiro 2003). To evaluate the effects of habitat type and sampling month on bacterial abundance, a two-way ANOVA with Type III sum of squares was performed, as described above. For cyanobacterial abundance, non-parametric comparisons were conducted using the Kruskal–Wallis test, also as previously described.

### Cytometric fingerprinting

Flow cytometry data gated for bacterial or cyanobacterial populations were exported in flow cytometry standard (FCS). All fluorescence and scatter channels were transformed using the inverse hyperbolic sine function: *f(x) = asinh(x)*. Cytometric fingerprints were then generated using the PhenoGMM fingerprinting approach (Rubbens et al. 2021a), which is based on Gaussian mixture models (see supplementary material for more details). Bray–Curtis dissimilarities were calculated between cytometric fingerprints and a non-metric multidimensional scaling (NMDS) was performed using the *beta_div_fcm* function (Phenoflow v1.1.2; Props et al. 2016). Differences in multivariate dispersion among groups were tested using *betadisper*, followed by ANOVA (*anova*, stats). Permutational multivariate analysis of variance (PERMANOVA) was conducted with *adonis2* (vegan v2.6–10; Oksanen et al. 2025), using 9999 permutations. Phenotypic diversity was quantified as the Hill number of order 2 (D₂, Inverse Simpson index), using the *Diversity_gmm* function with 10 000 bootstrap replicates (Phenoflow). To assess whether phenotypic diversity differed among habitats and months, we conducted Kruskal–Wallis tests with Dunn’s *post hoc* pairwise comparisons as above.

### Preparation of amplicon libraries

Immediately after sampling, pre-filtered water samples (150 μm) were concentrated on 0.2 μm filters (Cytiva Whatman 47 mm; nuclepore polycarbonate track-etched membranes). For each sample, duplicate filters were generated by filtering 150 ml of water. Filters were stored at −70°C until nucleic acid extraction. After all sampling campaigns were completed, sample order was randomized at each step of library preparation to minimize potential batch effects. DNA and RNA were co-extracted with the Easy-DNA kit (Invitrogen), following Protocol #3 with minor modifications (Székely et al. 2013). An additional step was included for the physical disruption of cells prior to chemical lysis: filters were bead-beaten with 0.2 ml of 0.1 mm zirconia/silica beads (BioSpec Products) for 15 min. RNase treatment was omitted during the final step to preserve both DNA and RNA. Extracts from duplicate filters were pooled per biological sample and half of the volume of each extract was purified for RNA work using the RNeasy PowerClean Pro Cleanup Kit (QIAGEN) per the manufacturer’s instructions. DNA was purified from the remaining volume using the DNeasy PowerClean Pro Cleanup Kit (QIAGEN).

To eliminate residual DNA, RNA extracts were treated with DNase I (Amplification Grade, Invitrogen). Successful DNA removal was verified by PCR (35 cycles) using the same primers intended for downstream amplicon sequencing (see Supplementary Table S2 for primer sequences), followed by visualization on 1% agarose gels. If DNA was detected, the DNase treatment was repeated. After confirming DNA removal, RNA was reverse-transcribed to complementary DNA (cDNA) with random hexamer primers, using the RevertAid H Minus First Strand cDNA synthesis kit (Thermo Fisher Scientific). Resulting cDNA was used directly for PCR amplification.

Amplicon libraries targeting the bacterial 16S rRNA gene (V3–V4 region) and eukaryotic 18S rRNA gene (V4–V5 region) were generated based on the protocol of Vass et al. (2020). Primers 341F/805RN (based on Herlemann et al. 2011) and 574F/1132R (Hugerth et al. 2014) were applied for 16S and 18S rRNA amplification, respectively. Four random nucleotides (NNNN) were included at the beginning of the forward primers of both genes to improve cluster detection on the Illumina flow cell during the initial sequencing cycles. In a first PCR, DNA and cDNA samples were amplified in triplicate reactions for each marker gene. The three technical replicates were pooled after amplification and sample-specific index sequences (barcodes) were introduced in a subsequent 15-cycle PCR (thermocycling conditions in Supplementary Table S3, Supplementary Table S4).

After each PCR step, amplicons were purified using magnetic beads (MagSi-NGSPREP Plus, magtivio). DNA concentrations were quantified with PicoGreen assays (Quant-iT PicoGreen dsDNA Assay Kit, Invitrogen) and equimolar amounts of each sample were pooled into a separate library for each gene. Libraries were further purified with the Wizard SV Gel and PCR Clean-Up System (Promega Corporation). Cluster generation and paired-end sequencing (2 × 300 bp) were performed using the Illumina MiSeq platform with MiSeq v3 chemistry. The 16S and 18S rRNA libraries were sequenced separately, with one flow cell used per gene.

### Processing of raw sequences

Primer sequences were removed from the raw reads using Cutadapt (v4.0 in Python v3.9.5; Martin 2011). Amplicon sequence variant (ASV) tables were constructed using the DADA2 pipeline (v1.28.0 in R v4.3.1; Callahan et al. 2016), following default parameters unless otherwise specified. For 16S rRNA, paired-end reads were merged with a minimum overlap of 16 nucleotides. Taxonomic classification was performed with a minimum bootstrap confidence threshold of 80, and the SILVA reference database (v138.1; Quast et al. 2013, Yilmaz et al. 2014) was used as the taxonomy training set. In the case of 18S rRNA, only forward reads were used as the paired-end reads did not overlap and the merging step was therefore omitted. Taxonomic assignment was carried out as in 16S rRNA, but with the PR2 reference database (v5.0.0; Guillou et al. 2012). The 18S rRNA ASV table was further curated with LULU (lulu v0.1.0; Frøslev et al. 2017).

Contaminant sequences were removed prior to downstream analyses. For 16S rRNA, chloroplast, mitochondrial, and unclassified sequences at the phylum level were excluded. For 18S rRNA, sequences assigned to Archaea, Bacteria, and eukaryotic plastids were removed, along with ASVs lacking supergroup-level classification. Additionally, sequences assigned to Rhodophyta, Metazoa, Fungi, and Embryophyceae were excluded to focus the analysis on microeukaryotic communities (Singer et al. 2021). Rarefaction curves were generated with *rarecurve* (vegan). For selected analyses, datasets were rarefied without replacement using the *rarefy_even_depth* function (phyloseq v1.50.0; McMurdie and Holmes 2013).

### Taxonomic diversity

Community composition of rRNA genes (“DNA”) and transcripts (“RNA”) was visualized using NMDS based on Bray–Curtis dissimilarity matrices, generated from rarefied 16S and 18S rRNA datasets (*ordinate* function with Bonferroni correction, phyloseq). To evaluate whether microbial communities clustered significantly by nucleic acid fraction, habitat type or sampling month, PERMANOVAs were performed as for cytometric fingerprints. Differences in the homogeneity of group dispersions were also assessed as above. Alpha diversity was estimated as observed richness (i.e. number of ASVs) using rarefied datasets, calculated separately for each nucleic acid fraction and habitat type. For 16S rRNA, differences in alpha diversity across habitats were assessed using Welch’s ANOVA tests, due to unequal variances, followed by Games–Howell *post hoc* comparisons (*anova_test* and *games_howell_test*; rstatix v0.7.2). For 18S rRNA, differences in richness across habitats were evaluated with Kruskal–Wallis tests, followed by Dunn’s *post hoc* tests, as described above. To visualize fluctuations in community composition, ASV counts from non-rarefied datasets were transformed into relative abundances. These were summarized at the phylum (16S rRNA) and subdivision levels (18S rRNA) for both DNA and RNA fractions of each biological sample.

To evaluate the extent to which cytometric diversity reflected bacterial taxonomic composition, Bray–Curtis dissimilarity matrices were computed from cytometric fingerprints for both bacterial and cyanobacterial populations. These matrices were then compared to those derived from 16S rRNA amplicon sequencing (DNA and RNA rarefied datasets), using Mantel tests (*mantel*, vegan) with 9999 permutations and Kendall’s rank correlation. For cyanobacterial comparisons, the 16S rRNA dataset included only ASVs under the phylum Cyanobacteria. For alpha diversity comparisons, the Inverse Simpson index was calculated for both the rarefied 16S rRNA datasets and for phenotypic diversity. The correlation between taxonomic and cytometric alpha diversity was evaluated using Kendall’s rank correlation (ggpubr v0.6.0).

Rank abundance plots were generated from rarefied datasets. Two versions were produced: one in which ASVs with identical relative abundances were assigned the same rank, and another in which each ASV was assigned a unique, sequential rank. Based on these plots, ASVs were categorized into three abundance groups: dominant, subdominant, and rare (Crevecoeur et al. 2022). Occupancy plots were also created per domain (i.e. bacteria and microeukaryotes) using non-rarefied datasets to illustrate ASV distribution across sequenced samples.

### RNA: DNA ratios and handling of phantom taxa

Phantom taxa, defined as ASVs with reads in the RNA fraction, but zero counts in the DNA fraction, were identified per biological sample using non-rarefied ASV tables for 16S and 18S rRNA datasets. Non-rarefied data were used in order to avoid misclassifying ASVs as phantom due to potential loss of DNA reads during rarefaction. All remaining ASVs with detectable DNA reads were classified as either active or inactive (Table 1). For each biological sample, the relative abundance of active, inactive, and phantom taxa was calculated separately for bacteria and microeukaryotes, and the taxonomic composition of these activity-inferred groups was visualized at the phylum (16S rRNA) and subdivision (18S rRNA) levels. To assess whether phantom taxa were also present at a coarser taxonomic resolution, bacterial and eukaryotic sequences were clustered into operational taxonomic units (OTUs) at 97% similarity (*Clusterize*, DECIPHER v3.2.0; Wright 2016).

**Table 1:** Classification criteria for ASVs from the 16S and 18S rRNA datasets based on activity status. Phantom taxa are defined by the exclusive presence of RNA reads, while active and inactive taxa are distinguished by the presence or absence of RNA reads among ASVs with detectable DNA.

RNA count	DNA count	Classification
>0	0	Phantom taxa
≥0	>0	Non-phantom taxa
>0	>0	Active taxa
0	>0	Inactive taxa

Phantom taxa yield undefined RNA: DNA ratios due to a denominator of zero (Bowsher et al. 2019). To address this, a pseudocount of 1 was added to all DNA entries in the ASV tables (Kearns et al. 2016). DNA and RNA read counts were then normalized by sequencing depth within each biological sample, and RNA: DNA ratios were calculated for each ASV by dividing RNA by DNA reads. To compare RNA: DNA ratios between phantom and non-phantom taxa, each ASV in each biological sample was treated as an independent observation. We used non-parametric bootstrapping (10 000 resamples) to compute the ratio of group means and a 99% confidence interval was derived from the 0.5^th^ and 99.5^th^ percentiles of the bootstrap distribution. A two-tailed *P* value for the null hypothesis (that the ratio equals 1) was calculated based on the proportion of bootstrap ratios ≤1.

### rRNA-based activity across lake transects

To investigate whether RNA: DNA ratios exhibited consistent spatial patterns among taxa shared across hydrologically connected sites, the ratios were extracted along four transects, one per lake, spanning from the main inlet, through two pelagic sites, to the corresponding outlet. For lake Stora Hålsjön, the closest site of the connecting stream was used as the outlet (see Supplementary Table S5 for transect details). To avoid overestimating activity, phantom taxa were excluded from this analysis. Due to the high variability in RNA: DNA ratios, Z-score normalization was applied to facilitate comparison across ASVs and sites. The standardized RNA: DNA ratios were visualized using heatmaps (*Heatmap*; ComplexHeatmap v2.22.0; Gu et al. 2016, Gu 2022). In addition, we assessed shifts in ASV abundance based on the abundance groups (dominant, subdominant, rare). ASVs that changed abundance category across a transect were classified as “conditionally rare” (Shade et al. 2014). Similarly, ASVs that changed activity category (active, inactive) across sites were classified as “shifting”. To further explore spatial and temporal patterns in RNA: DNA ratios, we identified ASVs present in multiple transects and/or sampling months. For each of these ASVs, we determined the habitat type (inlet, pelagic, or outlet) in which RNA: DNA ratios were highest within each transect and month. We then assessed whether these peaks were consistently associated with a particular habitat type or varied across sites.

### Community spatial turnover and assembly processes

To exemplify the spatial turnover of microbial communities towards the catchment outlet, we analyzed a transect in the study system spanning all sampled habitat types from the connecting stream (Hålsjöbäcken) to the outlet of lake Siggeforasjön. For each sampling month and for both 16S and 18S rRNA, we included all ASVs detected in both DNA and RNA fractions. At the most upstream site, all ASVs were considered to represent 100% of the community. At each downstream site, we calculated the total number of ASVs, the proportion shared with upstream sites and those unique to that location. This stepwise, cumulative comparison continued downstream to the catchment outlet. Additionally, we assessed the potential upstream origin of ASVs at the outlet by calculating the proportion shared with any upstream site and those unique to the outlet, for each month and domain.

For all DNA and RNA communities, assembly processes were inferred using the iCAMP framework (iCAMP v1.5.12; Ning et al. 2020). Details for phylogenetic trees are provided in the supplementary material. To assess the relative importance of ecological processes, we first calculated pairwise community turnovers between the catchment outlet and all pelagic samples for each month, separately for 16S and 18S rRNA. This allowed us to evaluate the extent of dispersal limitation at the catchment outlet across hydrologically distinct periods. Statistical differences in the relative contribution of dispersal limitation among months were tested using Kruskal–Wallis tests, followed by Dunn’s *post hoc* pairwise comparisons, as described above. We further compared assembly processes within habitats by analyzing pairwise turnovers within all streams (inlet, stream, outlet) and within pelagic habitats. For this, we grouped the relative contributions of homogenizing dispersal and dispersal limitation under “dispersal”, and both homogeneous and heterogeneous selection under “selection”. Differences in the relative importance of dispersal and selection processes between all streams and pelagic habitats were tested using Wilcoxon rank-sum tests (*wilcox.test*, stats).

## Results

### Environmental parameters

The model-estimated daily total water flow at the catchment outlet indicated that our sampling campaigns indeed captured distinct seasonal flow regimes: the early summer sampling occurred shortly after the high-flow period associated with snowmelt, the late summer sampling corresponded to the end of the driest period and the autumnal sampling took place during a period of moderate flow (Supplementary Fig. S1, Supplementary File 1). Modeled watercourse temperatures at the catchment outlet were similar for the two summer campaigns, while notably lower in autumn (Supplementary Fig. S2A). This aligned with our *in situ* measurements across all sampling locations, where water temperature (χ² (2) = 48.74, *P* < 0.001) and dissolved oxygen concentrations (χ² (2) = 29.80, *P* < 0.001) showed significant seasonal variation, with November having lower temperatures and higher oxygen levels than June and August (Supplementary Fig. S2B–C). In addition, vertical profiles of temperature and dissolved oxygen concentration in lakes indicated stratification during both summer campaigns, with complete mixing only in autumn (Supplementary Fig. S3). Two-way ANOVAs revealed that TOC, TN, and TP concentrations significantly differed among habitats, but showed no significant variation across months or their interaction (Supplementary Fig. S4, Supplementary Table S6). Specifically, inlets consistently exhibited significantly higher and more variable concentrations compared to pelagic environments, while no significant differences were observed between pelagic sites and outlets.

Flow cytometry measurements showed high precision, with a mean CV% in the counts of bacterial populations across samples of 0.70% (± 0.14% SD). A two-way ANOVA with Type III sums of squares revealed no statistically significant difference in natural log-transformed bacterial abundance across habitat types (F_3,42_ = 1.65, *P* = 0.193; Supplementary Fig. S5), sampling months (F_2,42_ = 2.80, *P* = 0.072), or their interaction (F_6,42_ = 0.96, *P* = 0.461). Cyanobacterial abundance did not differ significantly across habitats (Kruskal–Wallis χ² (3) = 5.72, *P* = 0.126), but showed a significant effect of month (χ² (2) = 12.70, *P* = 0.002). Dunn’s *post hoc* comparisons revealed significantly lower cyanobacterial abundance in November than in June, while differences between other months were not statistically significant (Supplementary Fig. S6).

### Rarefaction and abundance groups

All 54 biological samples were successfully sequenced for the 16S rRNA gene, yielding a total of 108 samples (DNA and RNA fractions). For the 18S rRNA gene, both DNA and RNA fractions were recovered from 46 samples, with an additional eight samples yielding only one fraction, resulting in a total of 100 sequenced samples (see Supplementary Table S7). Rarefaction curves plateaued, suggesting sufficient sequencing depth (Supplementary Fig. S7). The bacterial dataset initially contained 28 126 ASVs. After rarefaction to 20 500 reads per sample, 25 957 ASVs were retained. The microeukaryotic dataset comprised 6702 ASVs, with 5926 remaining after rarefaction to 11 800 reads.

Based on the shape of the rank abundance curves (Supplementary Fig. S8), ASVs were categorized into three abundance groups: “dominant” ASVs with a relative abundance ≥0.2%, “subdominant” ASVs between 0.002% and <0.2%, and “rare” ASVs with <0.002%. These thresholds were consistently applied to both bacterial and microeukaryotic datasets. Occupancy plots revealed that rare taxa exhibited consistently low occupancy across all sequenced samples. In contrast, subdominant and dominant ASVs displayed a broader range of occupancy values, with some occurring in only a few samples and others being widespread (Supplementary Fig. S9). Overall, 0.46% of bacterial ASVs were detected in at least half of the samples, compared to 0.78% for microeukaryotes.

### Taxonomic diversity

**Figure 2:**
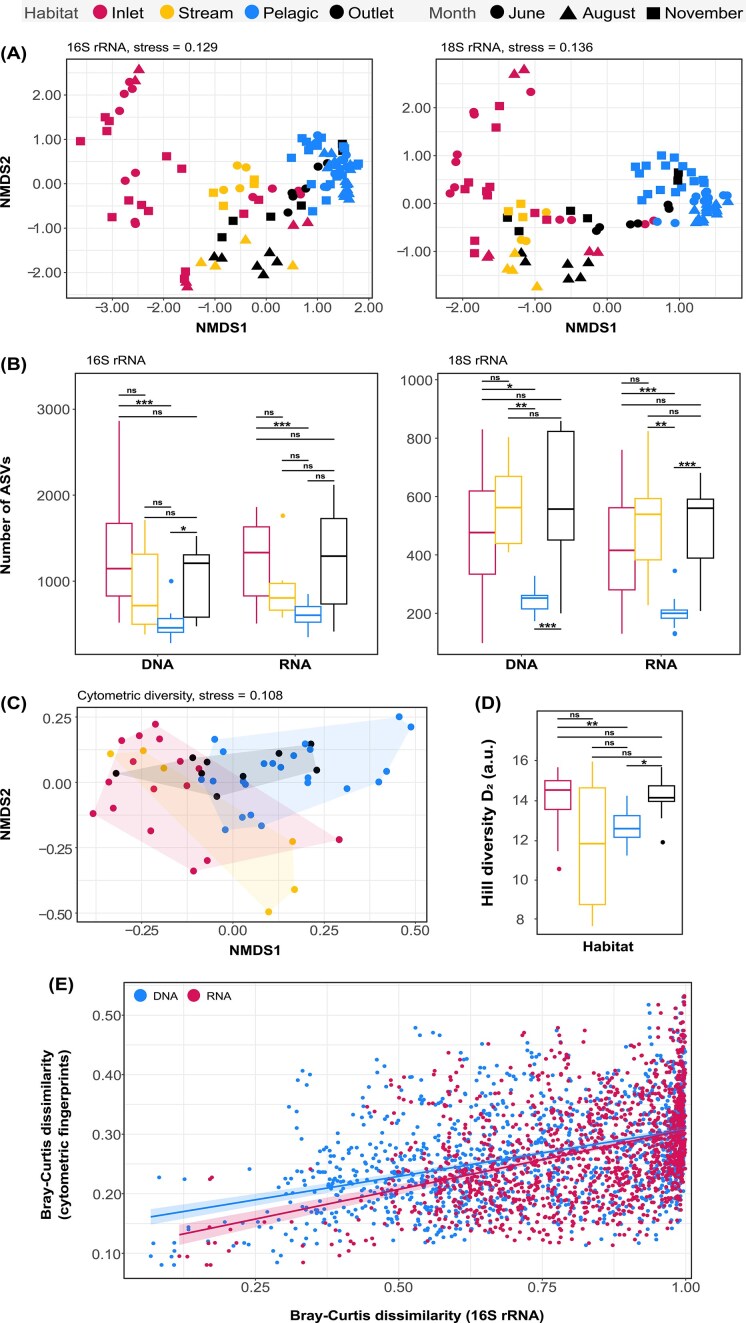
Taxonomic and cytometric diversity across habitats and sampling campaigns. (A) NMDS plots, showing bacterial (16S rRNA; left) and microeukaryotic (18S rRNA; right) community composition. Both DNA (rRNA genes) and RNA (rRNA transcripts) communities are included. Each symbol represents an individual sample (*n* = 108 for bacteria; *n* = 100 for microeukaryotes) with colors indicating habitat type and shapes representing the sampling month. (B) Observed species richness in bacterial (left) and microeukaryotic communities (right), shown by nucleic acid fraction. Boxplots are colored by habitat type. (C) NMDS plot of cytometric fingerprints from bacterial populations. Each symbol represents an individual sample (*n* = 54), colored by habitat type. (D) Phenotypic diversity of bacterial communities, expressed as Hill diversity D_2_ (arbitrary units), calculated from cytometric fingerprints. Boxplots are colored by habitat type. (E) Relationship between Bray–Curtis dissimilarities derived from cytometric fingerprints and 16S rRNA-based taxonomic profiles per nucleic acid fraction. Significance levels: *** *P* < 0.001; ** *P* < 0.01; * *P* < 0.05; ns = not significant.

NMDS plots revealed that DNA and RNA fractions from the same biological sample clustered closely together in ordination space (Supplementary Fig. S10), but PERMANOVA showed statistically significant yet small differences in community structure between DNA and RNA communities (bacteria: F_1,106_ = 5.24, R^2^ = 0.047, *P* < 0.001; microeukaryotes: F_1,98_ = 3.51, R^2^ = 0.035, *P* < 0.001). Samples also clustered by sampling month and habitat type (Fig. 2A), with PERMANOVA confirming significant effects of month, habitat, and their interaction on both bacterial and microeukaryotic community composition (*P* < 0.001; Supplementary Table S8, Supplementary Table S9). Habitat type explained a comparatively larger proportion of variation (bacteria: R^2^ = 0.211; microeukaryotes: R^2^ = 0.143) than month (bacteria: R^2^ = 0.057; microeukaryotes: R^2^ = 0.087). Lake pelagic communities consistently clustered closely in ordination space, whereas non-pelagic communities were more dispersed. For bacteria, *betadisper* indicated significant differences in dispersion among habitats (F_3,104_ = 67.34, *P* < 0.001), with pelagic communities differing from inlets (*P* < 0.001) and outlets (*P* = 0.002), but not the connecting stream (*P* = 0.997). A similar pattern was observed for microeukaryotes (F_3,96_ = 15.47, *P* < 0.001): pelagic zones differed significantly in dispersion from inlets (*P* < 0.001), but not from outlets (*P* = 0.105) or the stream (*P* = 0.323). Notably, outlet samples from the driest month (August) were consistently more distinct from pelagic samples in ordination space, a pattern observed for both domains.

ASV richness varied significantly across habitats for both bacterial and microeukaryotic communities, in both DNA and RNA fractions (Fig. 2B). For bacteria, Welch’s ANOVA on natural log-transformed richness revealed significant habitats differences (DNA: F_3,14.68_ = 16.73, *P* < 0.001; RNA: F_3,14.63_ = 10.7, *P* < 0.001). Games-Howell *post hoc* tests showed that pelagic DNA richness was lower than inlets (*P* < 0.001) and outlets (*P* = 0.02), with no differences among other habitats (*P* > 0.05). For RNA, pelagic richness was significantly lower than inlets (*P* < 0.001), but similar to outlets and the connecting stream (*P* > 0.05). For microeukaryotes, Kruskal–Wallis tests revealed significant habitat effects (DNA: χ² (3) = 22.08, *P* < 0.001; RNA: χ² (3) = 27.30, *P* < 0.001), with Dunn’s *post hoc* tests showing that pelagic sites had lower richness than all stream types (DNA: *P* < 0.05; RNA: *P* < 0.01). Overall, for microeukaryotes, pelagic environments had significantly lower richness than all stream types, while for 16S rRNA, only inlets had higher richness in both DNA and RNA fractions.

The relative abundance of bacterial phyla and eukaryotic subdivisions is shown in Supplementary Files 2 and 3 (A, B), respectively. All bacterial phyla and eukaryotic subdivisions detected in the dataset were present in both DNA and RNA fractions.

### Bacterial cytometric diversity

NMDS ordination and PERMANOVA of bacterial cytometric fingerprints (Fig. 2C) showed significant effects of sampling month (F_2,51_ = 2.53, *P* < 0.05), habitat type (F_3,50_ = 5.23, *P* < 0.001) and their interaction (F_11,42_ = 3.36, *P* < 0.001; Supplementary Table S10). The effect of habitat was substantial (R^2^ = 0.239), but the strongest effect was observed for the interaction between month and habitat type (R^2^ = 0.468). A Kruskal–Wallis test showed significant differences in the number of phenotypes across habitats (χ² (3) = 14.63, *P* = 0.002; Fig. 2D). The connecting stream locations exhibited greater variability in the number of phenotypes estimated by flow cytometry compared to other habitat types. Dunn’s *post hoc* tests showed that inlet and outlet streams had significantly higher phenotypic diversity than pelagic samples (*P* < 0.05), while no other pairwise comparison was significant (*P* > 0.05). Phenotypic diversity also differed among months (Kruskal–Wallis; χ² (2) = 8.09, *P* < 0.05; Supplementary Fig. S11), with November higher than June (*P* < 0.05).

Moderate but significant positive correlations were observed between cytometric and 16S rRNA-based Bray-Curtis dissimilarities for DNA (Mantel r = 0.3029, *P* < 0.001) and RNA fractions (Mantel r = 0.2838, *P* < 0.001), showing that phenotypic and taxonomic profiles captured overlapping patterns of beta diversity (Fig. 2E). Additional Mantel tests revealed significant, though weaker, correlations for cyanobacterial populations (DNA: r = 0.1988, *P* < 0.001; RNA: r = 0.1792, *P* < 0.001; Supplementary Fig. S12A). For alpha diversity of total bacterial populations, Kendall’s rank correlation tests showed moderate, significant associations between cytometric and 16S rRNA-based diversity, measured using the Inverse Simpson index. Significant correlations were observed for both DNA (R = 0.40, *P* < 0.001) and RNA fractions (R = 0.39, *P* < 0.001; Supplementary Fig. S12B), indicating that phenotypic and taxonomic approaches also captured similar trends in within-sample diversity.

### rRNA-based activity and phantom taxa

**Figure 3:**
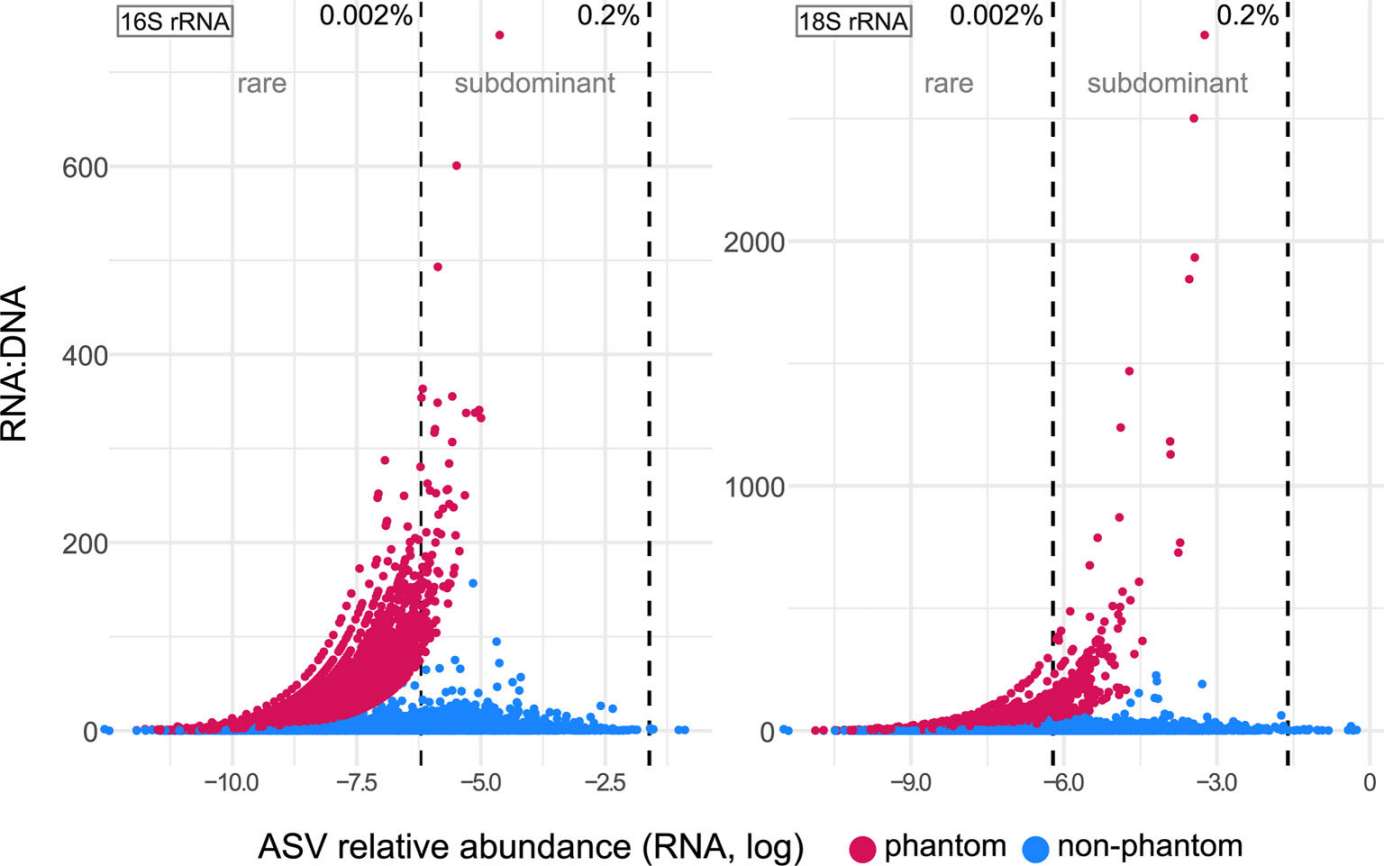
RNA: DNA ratios of phantom and non-phantom (active or inactive) ASVs across all biological samples for bacteria (16S rRNA; left) and microeukaryotes (18S rRNA; right). ASVs are grouped by their relative abundance in the RNA fraction into three categories: dominant (≥ 0.2%), subdominant (0.002%–< 0.2%,) and rare (< 0.002%). Vertical lines indicate the thresholds separating these groups. The *x*-axis is log-transformed (natural logarithm) to improve visualization of low-abundance ASVs.

Phantom taxa were consistently detected across all biological samples for both bacteria and microeukaryotes. The median proportion of phantom ASVs per biological sample was higher for bacteria (34.43%) compared to microeukaryotes (23.87%). Clustering sequences into OTUs did not substantially reduce these proportions (Supplementary Table S11). Across all ASV occurrences, active and inactive taxa spanned all abundance categories (Fig. 3). On the other hand, phantom taxa were associated with the subdominant and rare abundance groups, with the vast majority falling into the rare category. Phantom taxa exhibited disproportionately higher RNA: DNA ratios compared to non-phantom ASVs. For bacteria, the mean ratio was 16.27 (99% CI: 15.75–16.80; *P* < 0.001) and for microeukaryotes it was 18.44 (99% CI: 16.59–20.60; *P* < 0.001), indicating that phantom taxa may appear artificially more active than non-phantom ASVs when activity is inferred from RNA: DNA ratios, rather than reflecting increased biological activity.

On average, active bacterial taxa exhibited a relative abundance of 42% (±11 SD), followed by 33% (±12.63) phantom and 25% (±14.04) inactive. Similarly, active microeukaryotic taxa dominated with 44% (±8), followed by 34% (±10) inactive and 21% (±9) phantom. The relative abundance of bacterial phyla and microeukaryotic subdivisions is visualized for each activity-inferred subcommunity across biological samples (Supplementary Files 2C–F, 3C–F). Taxonomic profiles showed greater consistency within active, inactive and phantom groups than in the total DNA and RNA fractions.

### RNA: DNA ratios across lake transects

**Figure 4:**
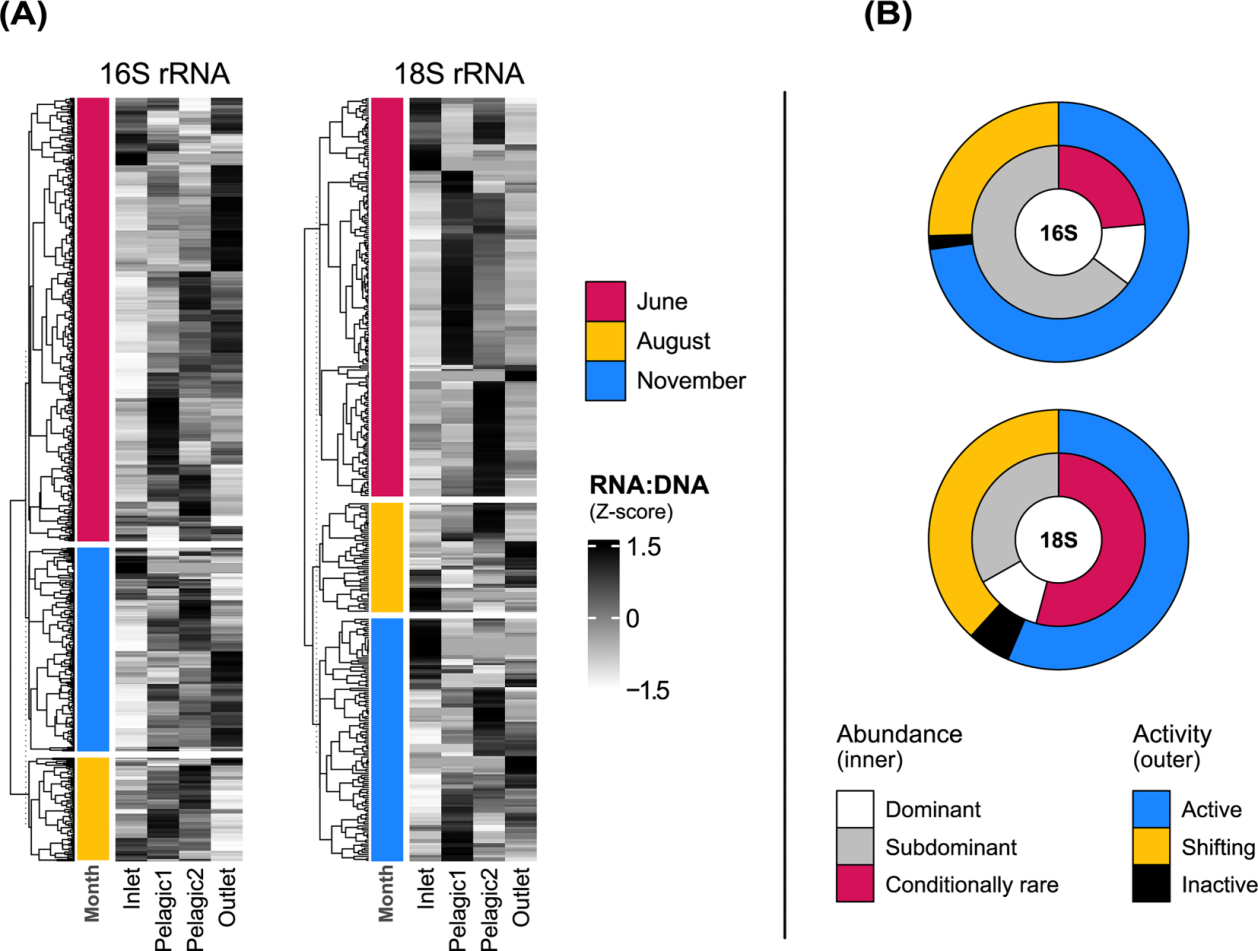
Spatiotemporal patterns of rRNA-activity and abundance at the ASV level across four lake transects (16S rRNA: *n* = 645; 18S rRNA: *n* = 371). (A) Heatmaps showing RNA: DNA ratios of shared bacterial (left) and microeukaryotic (right) ASVs across four hydrologically connected sites (inlet, two pelagic zones, outlet). Rows represent individual ASVs and data are grouped by sampling month. RNA: DNA ratios were Z-score standardized per ASV across locations within each transect. ASVs with RNA: DNA = 0 at all sites were excluded. (B) Circular diagrams summarizing abundance (inner rings) and activity (outer rings) classifications for the ASVs shown in (A).

The number of ASVs shared across lake transects varied seasonally, reflecting shifts in hydrological connectivity. For both domains, the fewest shared ASVs occurred in August, the driest month, followed by November and June (Fig. 4A). Relative abundances of shared ASVs at the level of bacterial phyla and eukaryotic subdivisions remained largely consistent across sampling campaigns (Supplementary Fig. S13), although the number of shared ASVs within each transect varied markedly among lake systems (Supplementary Table S12). Bacterial ASVs detected along transects spanned all abundance categories (Fig. 4B). However, all rare ASVs exhibited conditional rarity, becoming dominant or subdominant at least once per transect. In contrast, only dominant and subdominant ASVs were observed for microeukaryotes. Most microeukaryotic ASVs were also conditionally rare, shifting between dominant and subdominant within transects. Regarding activity patterns for both domains, the majority of shared ASVs were active and a substantial number exhibited shifting activity, alternating between active and inactive states across transect sites. A small number of ASVs were consistently inactive (RNA: DNA = 0 across all transect sites) for both bacteria (*n* = 11) and microeukaryotes (*n* = 21).

Finally, we examined whether RNA: DNA ratios of shared ASVs consistently peaked in the same habitat type across different transects and/or sampling months. Among bacterial ASVs, 139 were detected in more than one lake transect or month. Of these, two ASVs consistently showed highest RNA: DNA ratios in inlet habitats, 24 in pelagic zones and 13 in outlets. The majority (100 ASVs) exhibited variable habitat preferences, with the site of highest RNA: DNA ratio shifting across space or time. For microeukaryotes, 97 ASVs were detected in more than one transect or month. Of these, five showed consistently highest ratios in inlets, 35 in pelagic habitats, and one in outlets. The remaining 56 ASVs showed variable peak habitats. Overall, we did not observe a consistent increase or decrease of RNA: DNA ratios along the inlet–pelagic–outlet transects. Complete lists of ASVs with consistent or variable habitat-specific RNA: DNA peaks, along with taxonomic classifications, are provided in Supplementary Files 4 (bacteria) and 5 (microeukaryotes).

### Spatial turnover towards the catchment outlet

**Figure 5:**
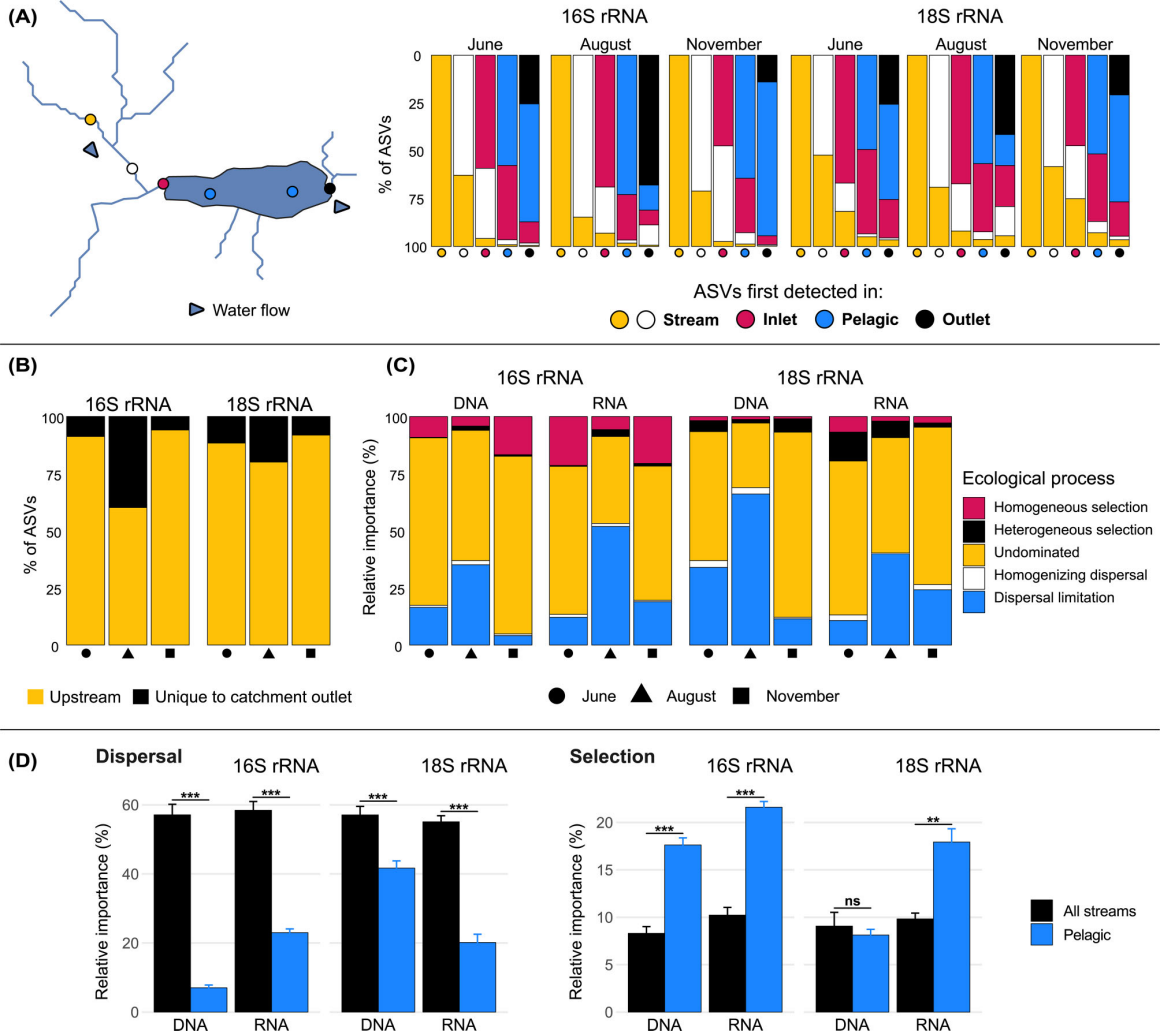
Spatial turnover towards the catchment outlet and assembly processes. (A) Spatial turnover of microbial communities across sampling locations in the most downstream lake (Siggeforasjön). Left: map with transect points and arrows indicating water flow towards the catchment outlet. Right: bar plots showing the proportion of ASVs retained or unique at each downstream site, relative to the immediately upstream location. (B) Proportion of ASVs detected at the catchment outlet that were also found in upstream locations or were unique to the outlet. (C) Relative contribution of ecological processes based on pairwise turnovers between the catchment outlet and all pelagic lake sites in the system. (D) Relative importance of dispersal and selection processes within habitats, comparing all stream (inlet, connecting stream, outlet) and pelagic turnovers. Bars represent the mean ± standard error. For all panels, data are shown separately for 16S and 18S rRNA genes. Colors represent sampling locations (A, B) or ecological processes (C, D); shapes indicate sampling months (B, C). Panels A and B consider all ASVs detected in both DNA and RNA fractions.

We observed consistent patterns of community turnover along the transect crossing the downstream lake towards the catchment outlet for both bacteria and microeukaryotes (Fig. 5A). Considering ASVs detected in both DNA and RNA fractions, each site harbored a combination of unique ASVs, first observed at that location, and shared ASVs that were also detected at upstream sites. At the catchment outlet, more than 50% of ASVs had already been detected at upstream locations along the transect, except for bacteria in August, where over half of the ASVs were unique to the outlet. This pattern was mirrored in the proportion of ASVs at the catchment outlet that were either detected across all sampling locations within the catchment or were exclusive to the outlet (Fig. 5B). The highest proportion of unique ASVs at the outlet of the system was again observed in August for both domains.

### Community assembly processes

Assembly processes were inferred with iCAMP for bacteria and microeukaryotes, comprising 387 and 93 phylogenetic bins, respectively. Dispersal limitation emerged as a dominant process for both microbial domains at the catchment outlet, with its relative contribution ranging from 4.12%–66.09% (Fig. 5C). This was followed by homogeneous (0.96%–21.41%) and heterogeneous selection (0.23%–12.64%), with the latter showing a higher relative importance in the 18S rRNA dataset. Homogenizing dispersal played a comparatively minor role in community assembly (0.32%–2.97%). Undominated processes (including stochastic drift, diversification, weak selection, and/or weak dispersal) accounted for 28.27%–80.99%.

Dispersal limitation varied seasonally at the catchment outlet, as revealed by Kruskal–Wallis tests (bacteria: *n* = 46; χ² (2) = 24.87, *P* < 0.001; microeukaryotes: *n* = 44; χ² (2) = 18.06, *P* < 0.001). Dunn’s *post hoc* tests showed significantly higher dispersal limitation in August compared to both June (bacteria: *P* < 0.001; microeukaryotes: *P* < 0.01) and November (bacteria: *P* < 0.001; microeukaryotes: *P* <0.001), while no significant difference was found between June and November (bacteria: *P* =1.00; microeukaryotes: *P* =1.00). Wilcoxon rank-sum tests showed that dispersal processes (dispersal limitation and homogenizing dispersal) were significantly more important in stream habitats for both domains and nucleic acid fractions. In contrast, selection processes (homogeneous and heterogeneous selection) were significantly more important in pelagic samples, except for the microeukaryotic DNA fraction (Fig. 5D; Supplementary Table S13).

## Discussion

In this study, we explored microbial diversity and community assembly within an aquatic network in central Sweden. Through detailed sampling across four lakes and their connecting streams, we assessed how bacterial and microeukaryotic communities responded to varying hydrological conditions. Using amplicon sequencing of both rRNA genes and transcripts, our findings build on previous work on spatiotemporal activity dynamics of bacterial communities by concurrently analyzing patterns in microbial eukaryotes. We found that microeukaryotes exhibited diversity and dispersal patterns similar to those of bacteria, with both groups strongly shaped by habitat-specific responses and, to a lesser extent, by seasonal variation.

### Pelagic zones exhibited lower richness than inlet streams

Taxonomic richness declined from inlets to pelagic habitats, in line with prior reports of higher richness in soils, soil water, and streams for both bacteria and microeukaryotes (Tamames et al. 2010, Crump et al. 2012, Ruiz-González et al. 2015, Sieber et al. 2020, Walters and Martiny 2020, Singer et al. 2021). A finding of greater novelty was that bacterial phenotypic richness, estimated via flow cytometric fingerprints, was also higher in inlets and outlets compared to lakes. Shallow, nutrient-rich inlets likely harbor diverse soil-derived taxa (Stadler and del Giorgio 2021), whereas the longer WRT in lakes may limit resources, constraining richness (Van der Gucht et al. 2007, Locey and Lennon 2019). Inlet-derived cells entering lakes may also get diluted or fail to establish under unfavorable conditions during or after dispersal (Lindström and Bergström 2004, Lindström and Langenheder 2012, Adams et al. 2014). Overall, our first hypothesis was supported for microeukaryotes, as pelagic environments exhibited significantly lower richness than all stream types, but only partly for bacteria, where both taxonomic and cytometric diversity were consistently higher only in inlet streams.

In terms of beta diversity, pelagic sites within lakes were highly similar regardless of stratification, indicating laterally well-mixed communities (Crump et al. 2012, Adams et al. 2014). Stream communities, however, exhibited greater spatial heterogeneity, as shown previously (Crump et al. 2007, 2012, Niño-García et al. 2016, Crevecoeur et al. 2022), often linked to their stronger terrestrial influence (Stadler and del Giorgio 2021). In our study, some inlets were ephemeral and dried completely during the summer campaigns, potentially leading to massive inoculation of cells from surrounding soils upon rewetting. Furthermore, our null model analysis indicated that the relative contribution of community assembly mechanisms was habitat-dependent, with dispersal processes having a higher importance in streams and deterministic taxon selection in lakes, the latter except for the microeukaryotic DNA fraction.

### RNA: DNA ratios showed no consistent spatial pattern at the ASV level

We observed significant differences between DNA and RNA-based community structure, as previously shown for both 16S and 18S rRNA genes (Charvet et al. 2014, Klein et al. 2016, Stadler and del Giorgio 2021). Across samples, the relative abundance of major taxonomic groups (i.e. bacterial phyla and eukaryotic subdivisions) in DNA- and RNA-derived communities was more variable than within each activity group (active, inactive or phantom taxa). Active and inactive ASVs spanned all abundance categories, whereas phantom taxa were predominantly rare, which likely contributed to this pattern.

Focusing on taxa shared along inlet–pelagic–outlet transects, some ASVs consistently showed habitat-specific RNA: DNA peaks across transects and/or sampling months, whereas others displayed variable peak locations, as observed for the majority of bacterial ASVs. We also identified ASVs from both domains that were passively dispersed and remained inactive (RNA: DNA = 0) across all sites, as observed elsewhere (Mestre and Höfer 2021, Crevecoeur et al. 2022). Additionally, all rare bacterial ASVs shifted to dominant or subdominant across transects, supporting previous findings that rare taxa with restricted distributions (Liu et al. 2015) can be highly dynamic and responsive (Jones and Lennon 2010, Crevecoeur et al. 2022). It is important to note, however, that these analyses included only ASVs shared across transects, so low-occupancy taxa, which represented most of ASVs, could not be evaluated.

Overall, our results do not support our second hypothesis, as we did not observe a consistent relative increase or decrease of RNA: DNA ratios of shared taxa along lake transects. We speculate that this variability may reflect genuine ecological differences among microbial taxa, with ASVs responding differently across aquatic habitats. It may also reflect methodological or physiological factors, such as the compositionality of RNA and DNA sequencing data, which can influence RNA: DNA ratios independent of absolute activity, or differences in growth phase (Steven et al. 2017), as cells within the same ASV population can exhibit heterogeneous levels of rRNA transcription, complicating the interpretation of RNA: DNA patterns across habitats. Consequently, alternative approaches will be needed in future studies to further resolve microbial activity shifts along spatial gradients.

### Hydrology shaped community turnover at the catchment outlet

The number of shared ASVs in inlet–pelagic–outlet transects reflected the seasonal water flow dynamics, with the lowest overlap occurring during the driest period. The number of shared ASVs also varied across lakes, likely as a function of WRT (Lindström and Bergström 2004, Lindström et al. 2006). For instance, more bacterial ASVs were shared across sites in the downstream lake (Siggeforasjön) with 180 days WRT, than in lake Stora Hålsjön with 300 days (WRTs from Brunberg and Blomqvist 1998).

Bacterial and microeukaryotic communities showed clear turnover from the connecting stream to the pelagic zone of the downstream lake and ultimately to the catchment outlet, consistent with previous findings (Crump et al. 2012, Read et al. 2015, Ruiz-González et al. 2015). The catchment outlet community comprised taxa with distinct ecological histories, with some being unique to specific locations, while others being potentially progressively recruited along the hydrological gradient, a pattern also observed by Stadler and del Giorgio (2021) and Crevecoeur et al. (2022). This historical imprint, which varied with the prevailing flow regime, implied that bacteria and microbial eukaryotes may share similar dispersal dynamics in aquatic networks (Crump et al. 2012, Wu et al. 2018, Liu et al. 2020, Sun et al. 2021).

We lastly hypothesized that the catchment outlet would be more separated from upstream communities during the driest season. We fail to reject this hypothesis since our seasonal sampling captured more unique taxa compared to upstream habitats in August, for both bacterial and microeukaryotic communities. This was reflected in higher dispersal limitation from the pelagic environment for both DNA and RNA communities, as shown by the null model analysis. Previous studies suggest that when WRT is short, mass effects dominate (Lindström and Östman 2011, Niño-García et al. 2016), allowing inlet stream bacteria to mix farther into the lake (Adams et al. 2014). However, in our system, homogenizing dispersal appeared to have a very small relative importance (<3%), indicating that short WRT was rather associated with reduced dispersal limitation. Lastly, although the studied lake network is generally subject to low human impact, we cannot exclude that local recreational activities near the downstream lake (Siggeforasjön) and the catchment outlet, where a camping site is located, contributed to observed differences between pelagic and outlet communities, particularly in August.

### Methodological considerations of cytometric fingerprints

Flow cytometric diversity captures microbial spatiotemporal variability and correlates with the taxonomic diversity derived from 16S rRNA gene sequencing, with correlation strength varying by ecosystem type (Rubbens et al. 2021b, García et al. 2015, Props et al. 2016, 2018a,b, Coggins et al. 2020). Our results revealed moderate, statistically significant correlations between cytometric and taxonomic alpha and beta diversity metrics. Its speed, cost-effectiveness and minimal sample requirements, make flow cytometry well-suited for biogeographic studies by tracking broad-scale community shifts, assessing temporal variation or guiding sampling strategies during pilot studies. Cytometric profiles may also help predict the presence of abundant taxa (Heyse et al. 2021), enabling spatial extrapolation from a subset of sequenced samples. However, flow cytometry may exclude some particle-associated bacteria (particles larger than 40 μm in our study) and it captures broad phenotypic shifts, while sequencing provides finer taxonomic resolution based on relative gene abundances (García et al. 2015, Rubbens et al. 2019). Therefore, a perfect correlation between cytometric and taxonomic data is not expected.

### Methodological considerations of phantom taxa

Phantom taxa, although theoretically unexpected, are common in dual-fraction studies and the proportions we observed closely matched previous reports (Klein et al. 2016, Bowsher et al. 2019). Since most prior biogeography studies applying RNA: DNA ratios used OTUs (Stadler and del Giorgio 2021, Crevecoeur et al. 2022, Wu et al. 2024), we also clustered our sequences to 97% similarity, but this did not substantially alter the proportions. Possible causes for their existence include extraction and amplification bias, as well as incomplete sequencing of rare taxa, errors in cDNA synthesis and DNA contamination in RNA extracts (Gurp et al. 2013, Klein et al. 2016, Lim et al. 2016, Bowsher et al. 2019). We minimized these potential factors by co-extracting DNA and RNA, and verifying the complete degradation of DNA in RNA samples through a PCR test. In addition, our rarefaction curves demonstrated sufficient sequencing depth. PCR amplification may also play a role; while PCR replicates are generally consistent in dominant taxa, rare taxa are often recovered stochastically (Shirazi et al. 2021), and the vast majority of phantom taxa in our dataset were indeed rare.

Since phantom taxa are unavoidable when using RNA: DNA ratios of rRNA genes (Bowsher et al. 2019), various strategies have been proposed to handle them. Some studies exclude them entirely from analysis to infer biogeographic patterns (e.g. Crevecoeur et al. 2022), while others apply correction methods for the zero denominator before calculating RNA: DNA ratios (Denef et al. 2016, Kearns et al. 2016, Bowsher et al. 2019, Stadler and del Giorgio 2021 and this study). Despite correction, we show that phantom taxa still exhibited disproportionately high RNA: DNA ratios compared to non-phantom ASVs in both 16S and 18S rRNA datasets. Even the most extreme correction (i.e. setting phantom ratios to 100; Bowsher et al. 2019) would fail to capture all observed values, indicating that phantom taxa cannot be directly compared to non-phantom ones in terms of activity levels. Therefore, caution is warranted when comparing RNA: DNA ratios across taxa or spatiotemporal gradients, since failing to account for phantom taxa may make them appear more active than genuinely active taxa (with both RNA and DNA reads detected), potentially leading to erroneous ecological interpretations. A final key consideration is rRNA operon copy numbers, which vary across lineages and can bias RNA: DNA ratios, particularly in microeukaryotes where some taxa possess especially high 18S rRNA gene copy numbers (Lee et al. 2009, Louca et al. 2018, Gong and Marchetti 2019).

## Conclusions

Based on amplicon sequencing of both rRNA genes and transcripts, our study revealed covarying diversity patterns and similar dispersal capabilities between bacteria and microbial eukaryotes across a stream–lake network in Sweden. Inlet streams exhibited consistently higher taxonomic and bacterial cytometric diversity compared to pelagic habitats. In addition, bacterial and microeukaryotic communities were likely shaped by parallel community assembly processes, with dispersal having a greater relative importance in streams and environmental selection in pelagic environments. Notably, dispersal limitation emerged as an important structuring force at the catchment outlet during the dry season. From a methodological aspect, we addressed the detection of phantom taxa, which were primarily rare and exhibited disproportionately high RNA: DNA ratios compared to active taxa, highlighting the importance of cautious interpretation of rRNA-based activity metrics. Additionally, computational flow cytometry reliably captured biogeographic trends in bacterial communities, providing a scalable and complementary approach to sequencing. Future research should expand investigations of microeukaryotes into river networks and move beyond ribosomal genes to include functional markers and viral communities.

## Supplementary Material

fiag010_Supplemental_Files

## Data Availability

The raw sequencing data for the 16S rRNA genes and transcripts have been deposited in the European Nucleotide Archive (ENA) at EMBL-EBI under accession number PRJEB94279, while the 18S rRNA genes and transcripts under PRJEB94290. Flow cytometry data (.FCS files) are available in Zenodo, at https://doi.org/10.5281/zenodo.16309593. All other relevant data supporting the findings of this study are available within the article and its Supplementary material. Custom scripts developed for data analysis are available from the corresponding author upon reasonable request.
