# Supplementary material for: Shared stream–lake patterns in diversity, rRNA-based activity and community assembly of bacteria and microeukaryotes under distinct hydrological regimes
Source: FEMS Microbiol Ecol. 2026 Feb 11;102(3):fiag010. doi: 10.1093/femsec/fiag010 (PMC12923169; doi:10.1093/femsec/fiag010)
Supplement: fiag010_Supplemental_Files [file fiag010_supplemental_files.zip › Supplementary_File1.pdf]

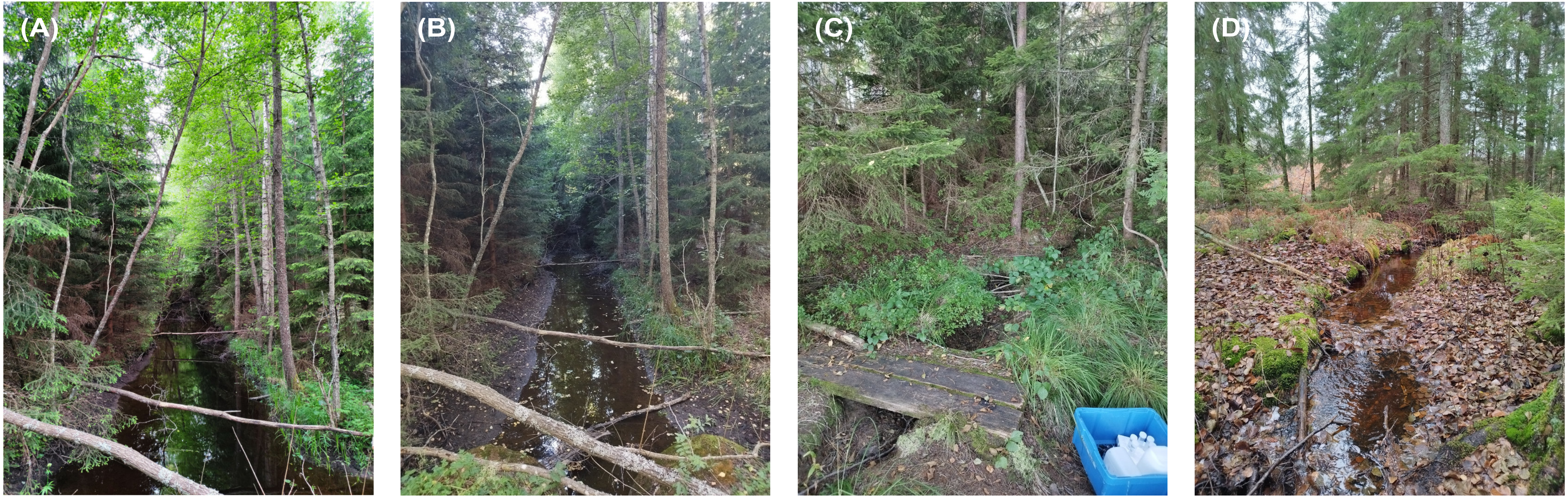

Supplementary File 1. Photos from selected sampling locations illustrating differences in hydrological regimes. (A) Main inlet of the downstream lake Siggeforasjön (SIG-Inlet1) in June (13 June 2022); water depth at the center of the stream was 43 cm. (B) The same inlet under dry conditions in August (25 August 2022); water depth at the same location was 24.3 cm. (C) An ephemeral inlet stream to lake Siggeforasjön (SIG-Inlet2) in August, when it was completely dry. (D) Main inlet of lake Tarmlången (TAR-Inlet), another ephemeral stream, shown with water flow during the sampling campaign in November (2 November 2022). Photos captured using Xiaomi smartphones.
