# Supplementary material for: Shared stream–lake patterns in diversity, rRNA-based activity and community assembly of bacteria and microeukaryotes under distinct hydrological regimes
Source: FEMS Microbiol Ecol. 2026 Feb 11;102(3):fiag010. doi: 10.1093/femsec/fiag010 (PMC12923169; doi:10.1093/femsec/fiag010)
Supplement: fiag010_Supplemental_Files [file fiag010_supplemental_files.zip › Supplementary_File3.pdf]

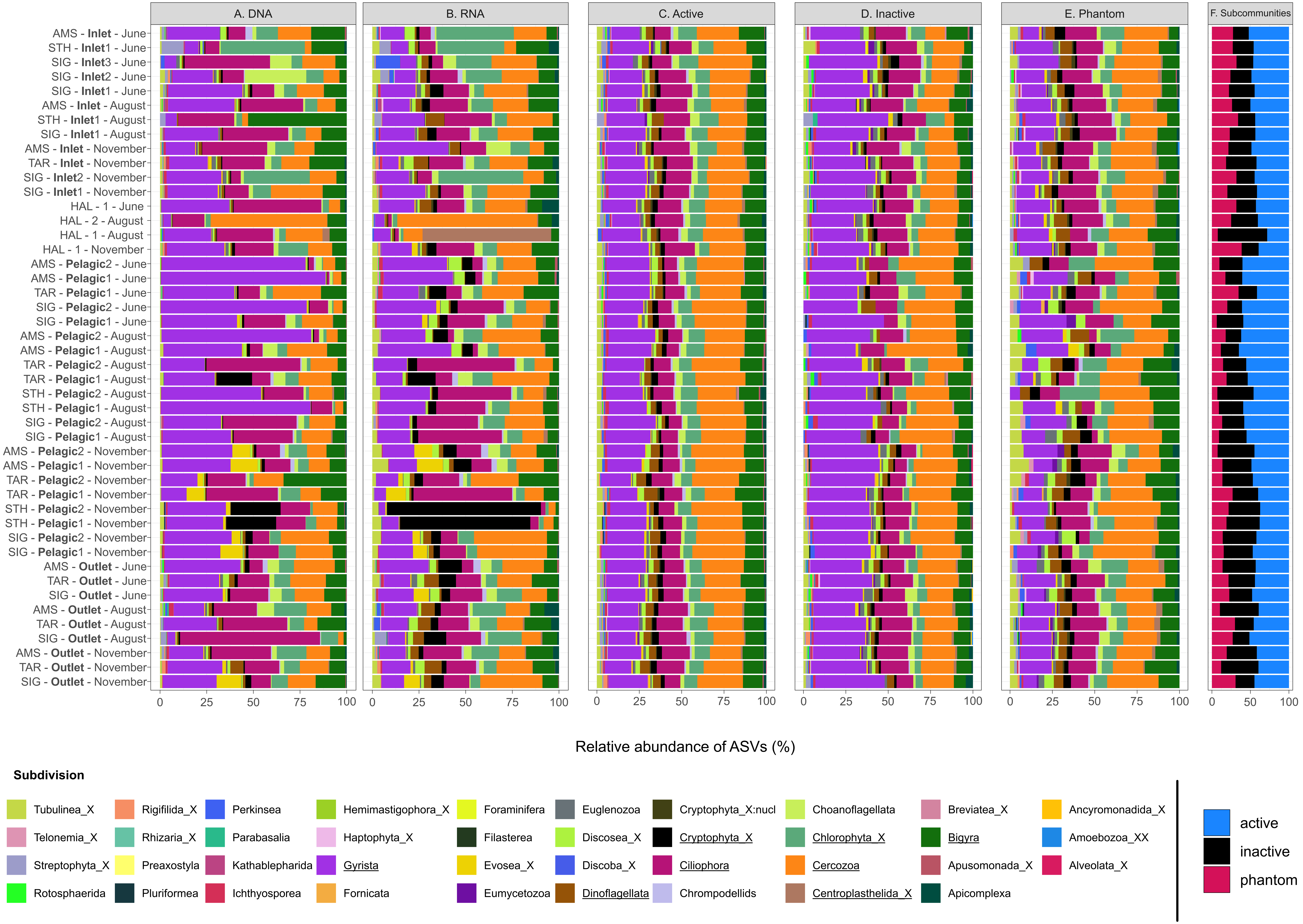

Supplementary File 3. (A–B) Relative abundance of Amplicon Sequence Variants (ASVs), grouped by subdivision, across all biological samples sequenced for the 18S rRNA gene. Panel A shows the DNA fraction (18S rRNA genes) and B the RNA fraction (18S rRNA transcripts). Each bar represents an individual sample, with colors indicating different subdivisions. The order of colors in the legend corresponds to their order in the bar plots from left to right. Abundant subdivisions are also underlined in the legend. (C–E) Relative abundance of ASVs grouped by subdivision within three subcommunities: active (DNA > 0 and RNA > 0), inactive (DNA > 0 and RNA = 0) and phantom (DNA = 0 and RNA > 0) taxa, shown per biological sample. (F) Proportion of active, inactive and phantom ASVs in each biological sample.
