# Supplementary material for: Shared stream–lake patterns in diversity, rRNA-based activity and community assembly of bacteria and microeukaryotes under distinct hydrological regimes
Source: FEMS Microbiol Ecol. 2026 Feb 11;102(3):fiag010. doi: 10.1093/femsec/fiag010 (PMC12923169; doi:10.1093/femsec/fiag010)
Supplement: fiag010_Supplemental_Files [file fiag010_supplemental_files.zip › Supplementary_material.docx]

**Supplementary data and methods for:**

**Shared stream****–lake patterns in diversity, rRNA-based activity and community assembly of bacteria and microeukaryotes under distinct hydrological regimes**

Sofia Papadopoulou*, Eva S. Lindström

Department of Ecology and Genetics/Limnology, Uppsala University, Norbyvägen 18 D, 752 36 Uppsala, Sweden

*Corresponding author: [sofia.papadopoulou@ebc.uu.se](mailto:sofia.papadopoulou@ebc.uu.se)

*Supplementary methods: Sample locations*

**Table S1.** List of the 54 samples collected across the catchment area during the three seasonal sampling campaigns. System abbreviations: AMS: lake Ämsjön, STH: lake Stora Hålsjön, HAL: Hålsjöbäcken ("connecting stream"), TAR: lake Tarmlången, SIG: lake Siggeforasjön. Sampling months are abbreviated as J: June, A: August and N: November. Coordinates are provided in WGS84 format.

| **Sample** | **System** | **Habitat type** | **Month** | **Latitude** | **Longitude** |
| --- | --- | --- | --- | --- | --- |
| AMS-Inlet | AMS | Inlet | J, A, N | 59.9554 | 17.0886 |
| AMS-Pelagic1 | AMS | Pelagic | J, A, N | 59.9595 | 17.0955 |
| AMS-Pelagic2 | AMS | Pelagic | J, A, N | 59.9628 | 17.1016 |
| AMS-Outlet | AMS | Outlet | J, A, N | 59.9711 | 17.1119 |
| STH-Inlet1 | STH | Inlet | J, A, N | 59.9896 | 17.0900 |
| STH-Inlet2 | STH | Inlet | J, N* | 59.9918 | 17.0973 |
| STH-Pelagic1 | STH | Pelagic | J, A, N | 59.9898 | 17.0935 |
| STH-Pelagic2 | STH | Pelagic | J, A, N | 59.9906 | 17.0990 |
| HAL-1 | HAL | Connecting stream | J, A, N | 59.9836 | 17.1146 |
| HAL-2 | HAL | Connecting stream | J, A, N | 59.9796 | 17.1222 |
| TAR-Inlet | TAR | Inlet | N* | 59.9766 | 17.0754 |
| TAR-Pelagic1 | TAR | Pelagic | J, A, N | 59.9790 | 17.1000 |
| TAR-Pelagic2 | TAR | Pelagic | A, N | 59.9763 | 17.0777 |
| TAR-Outlet | TAR | Outlet | J, A, N | 59.9758 | 17.1187 |
| SIG-Inlet1 | SIG | Inlet | J, A, N | 59.9772 | 17.1274 |
| SIG-Inlet2 | SIG | Inlet | J, N* | 59.9750 | 17.1421 |
| SIG-Inlet3 | SIG | Inlet | J, N* | 59.9745 | 17.1460 |
| SIG-Pelagic1 | SIG | Pelagic | J, A, N | 59.9769 | 17.1371 |
| SIG-Pelagic2 | SIG | Pelagic | J, A, N | 59.9768 | 17.1528 |
| SIG-Outlet | SIG | Outlet | J, A, N | 59.9779 | 17.1595 |

* These inlet streams were ephemeral (i.e. temporary streams that appeared only after rainfall or snowmelt) and were not sampled in all campaigns because they were dry.

*Supplementary methods: Total carbon, nitrogen and phosphorus*

Unfiltered water samples were collected in acid-washed plastic bottles for the analysis of total organic carbon (TOC), total nitrogen (TN) and total phosphorus (TP) concentrations. The bottles had been sequentially submerged in three separate baths: deionized water for 24 h, 5% HCl for 4 h and again in deionized water for 24 h. For TOC and TN, subsamples were transferred to 40 mL glass vials, acid-washed (deionized water for 24 h; 10% HCl for 24 h; deionized water for 24 h) and combusted in a muffle oven (4 h, 450°C), and stored at 4°C in the dark until analysis (within one week). TP samples were stored at -20°C and analyzed after the final sampling campaign.

TOC, measured as non-purgeable organic carbon, and TN were analyzed in instrumental triplicates with a Shimadzu, TOC-L/TNM-L (Kyoto, Japan). TOC calibration was performed using potassium hydrogen phthalate (KHP) and ethylenediaminetetraacetic acid (EDTA) was used as a secondary standard to check the calibration curve. For TN, calibration was done with potassium nitrate (KNO_3_). TP was determined photometrically at 882 nm (GENESYS 50 UV‑Vis spectrophotometer, Thermo Scientific) in triplicates using the molybdenum-blue method after oxidative hydrolysis with potassium persulfate in acidic solution at high temperature and pressure in an autoclave (Murphy and Riley 1958, Menzel and Corwin 1965).

*Supplementary methods: Cytometric fingerprinting*

All fluorescence and scatter channels in the exported FCS files (bacterial or cyanobacterial gated populations) were transformed using the inverse hyperbolic sine function: *f(x) = asinh(x)*. For beta diversity estimation, four channels were selected: FSC-A, SSC-A, FL1-A and FL3-A. Fluorescence intensities were normalized by dividing each parameter by the maximum observed SYBR Green I fluorescence value (FL1-A, transformed) across all samples. Kernel densities were computed using the *flowBasis* function (flowFDA v0.99). Samples were randomly subsampled to 7000 particles (matching the sample with the lowest bacterial cell count), and a 256 × 256 binning grid was created for each channel pair. Kernel density estimation (Gaussian kernel, bandwidth = 0.01) was performed for each bin. The resulting density values were concatenated into a one-dimensional vector representing the cytometric fingerprint. For cyanobacterial subpopulations, normalization was performed using the FL3-A channel. Subsampling was done at 862 particles per sample and 128 × 128 grids were generated.

Due to computational demands, alpha diversity estimates for bacterial communities (phenotypic diversity) were based on three parameters: FSC-H, SSC-H and FL1-H. The *PhenoGMM* function (Phenoflow v1.1.2 in R v4.3.1) was used to train a GMM-fitted model on 7000 subsampled cells per sample. Phenotypic diversity was quantified as the Hill number of order 2 (D₂, Inverse Simpson index), using the *Diversity_gmm* function (Phenoflow) with 10 000 bootstrap replicates (Props et al. 2016).

*Supplementary methods: Primers and PCR conditions*

**Table S2.** Primer sequences for the rRNA genes used in the study.

| **Primer** | **Sequence** |
| --- | --- |
| **16S rRNA (V3-V4)** |  |
| 341F | NNNNCCTACGGGNGGCWGCAG |
| 805R | GACTACNVGGGTATCTAATCC |
| **18S rRNA (V4-V5)** |  |
| 574f | NNNNCGGTAAYTCCAGCTCYAV |
| 1132r | CCGTCAATTHCTTYAART |

**Table S3.** PCR1 cycling conditions for 16S and 18S rRNA gene amplification.

| **16S rRNA** |  |  |  |
| --- | --- | --- | --- |
| **Step** | **Temperature** | **Time** |  |
| Denaturation | 98^o^C | 3 min |  |
| Denaturation | 98^o^C | 10 s | 20* cycles |
| Annealing | 48^o^C | 30 s |  |
| Extension | 72^o^C | 30 s |  |
| Final extension | 72^o^C | 2 min |  |

| **18S rRNA** |  |  |  |
| --- | --- | --- | --- |
| **Step** | **Temperature** | **Time** |  |
| Denaturation | 98^o^C | 30 s |  |
| Denaturation | 98^o^C | 10 s | 20* cycles |
| Annealing | 51^o^C | 30 s |  |
| Extension | 72^o^C | 45 s |  |
| Final extension | 72^o^C | 2 min |  |

* 20 cycles were used to test for DNA presence in extraction controls, while 35 cycles for residual DNA in DNase‑treated samples.

**Table S4.** PCR2 indexing conditions.

| **16S/18S rRNA** |  |  |  |
| --- | --- | --- | --- |
| **Step** | **Temperature** | **Time** |  |
| Denaturation | 98^o^C | 30 s |  |
| Denaturation | 98^o^C | 10 s | 15 cycles |
| Annealing | 66^o^C | 30 s |  |
| Extension | 72^o^C | 30 s |  |
| Final extension | 72^o^C | 2 min |  |

*Supplementary methods: ASV reactivity across lake transects*

**Table S5.** Overview of samples included in each lake transect for the assessment of microbial reactivity at the amplicon sequence variant (ASV) level. System abbreviations: AMS: lake Ämsjön, STH: lake Stora Hålsjön, HAL: Hålsjöbäcken ("connecting stream"), TAR: lake Tarmlången, SIG: lake Siggeforasjön. Sampling months are abbreviated as J: June, A: August and N: November. The names of the biological samples are further explained in Table S1.

| **Transect** | **System** | **Biological samples** | **Month (rRNA gene)** |
| --- | --- | --- | --- |
| 1 | AMS | AMS-Inlet, AMS-Pelagic1, AMS-Pelagic2, AMS-Outlet | J, A, N (16S/18S) |
| 2 | STH | STH-Inlet1, STH-Pelagic1, STH-Pelagic2, HAL-1 | J, A, N (16S)  A (18S*) |
| 3 | TAR | TAR-Inlet, TAR-Pelagic1, TAR-Pelagic2, TAR-Outlet | N (16S/18S) |
| 4 | SIG | SIG-Inlet1, SIG-Pelagic1, SIG-Pelagic2, SIG-Outlet | J, A, N (16S/18S) |

* 18S rRNA data for STH transect were only available for August due to missing DNA/RNA fractions in June and November for samples that were not successfully sequenced (see Table S7).

*Supplementary methods: Phylogenetic trees for iCAMP*

For both 16S and 18S rRNA datasets, sequence alignments were performed on non-rarefied data using the *AlignSeqs* function (DECIPHER v3.2.0; Wright 2016) with default parameters. Phylogenetic trees were constructed from untrimmed alignments using the ape (v5.7.1; Paradis, Claude and Strimmer 2004) and phangorn (v2.11.1; Schliep 2011) packages in R (v4.3.1). Specifically, pairwise distances were calculated under the F81 model (*dist.ml*, phangorn) and neighbor-joining (NJ) trees were estimated (*nj*, ape). These served as starting trees for maximum likelihood (ML) optimization (*pml* and *optim.pml*, phangorn) under a GTR + I + Γ substitution model, with stochastic NNI rearrangements. NJ was used to reduce computational burden given dataset size; final trees were fully optimized under ML. Final trees were then midpoint-rooted (*midpoint*, phangorn). Separately for bacteria and microbial eukaryotes and for all DNA and RNA communities, these trees were used to infer community assembly processes with the iCAMP framework (Ning et al. 2020), using the *icamp.big* function (iCAMP v1.5.12) with default settings and 1000 randomizations.

*Supplementary results: Water flow*


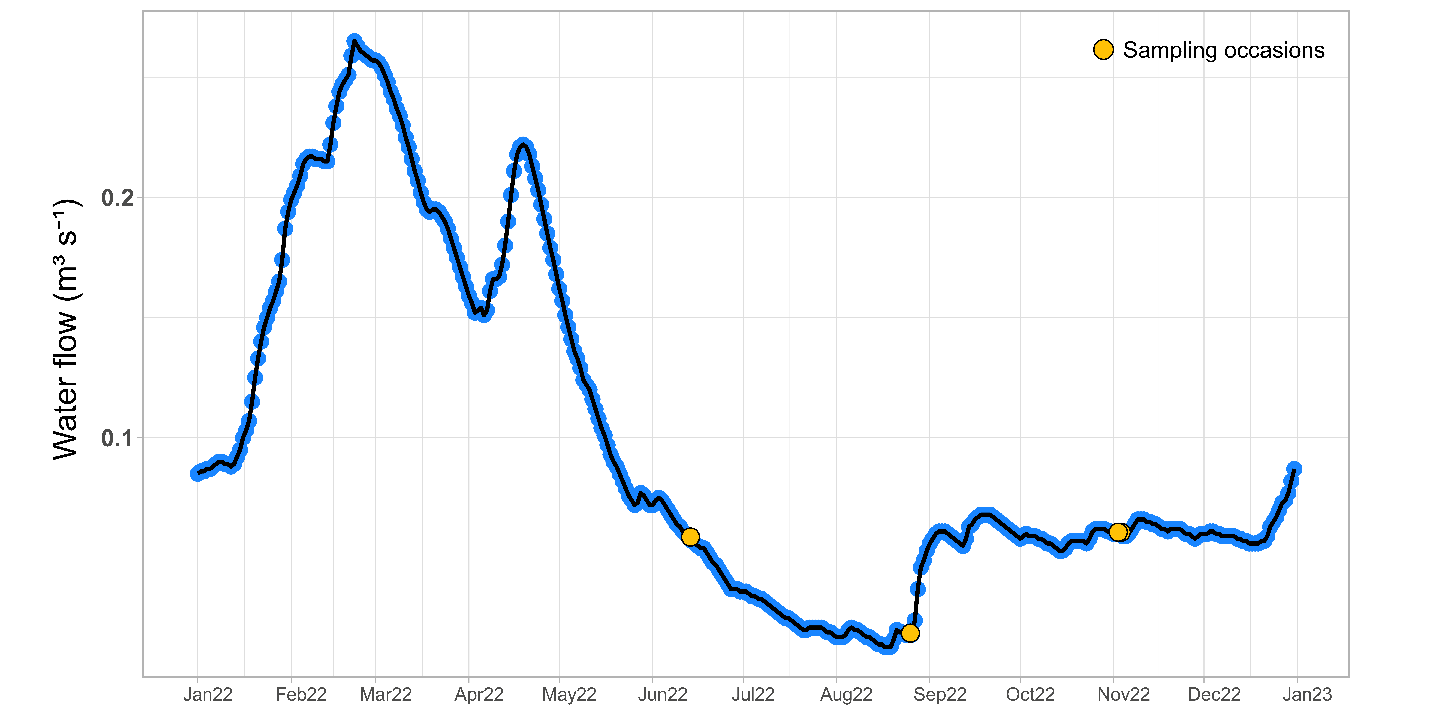


**Figure S1.** Model-estimated daily total water flow at the catchment outlet (outlet of lake Siggeforasjön) with the 2022 sampling occasions marked.

*Supplementary results: Water temperature and dissolved oxygen*


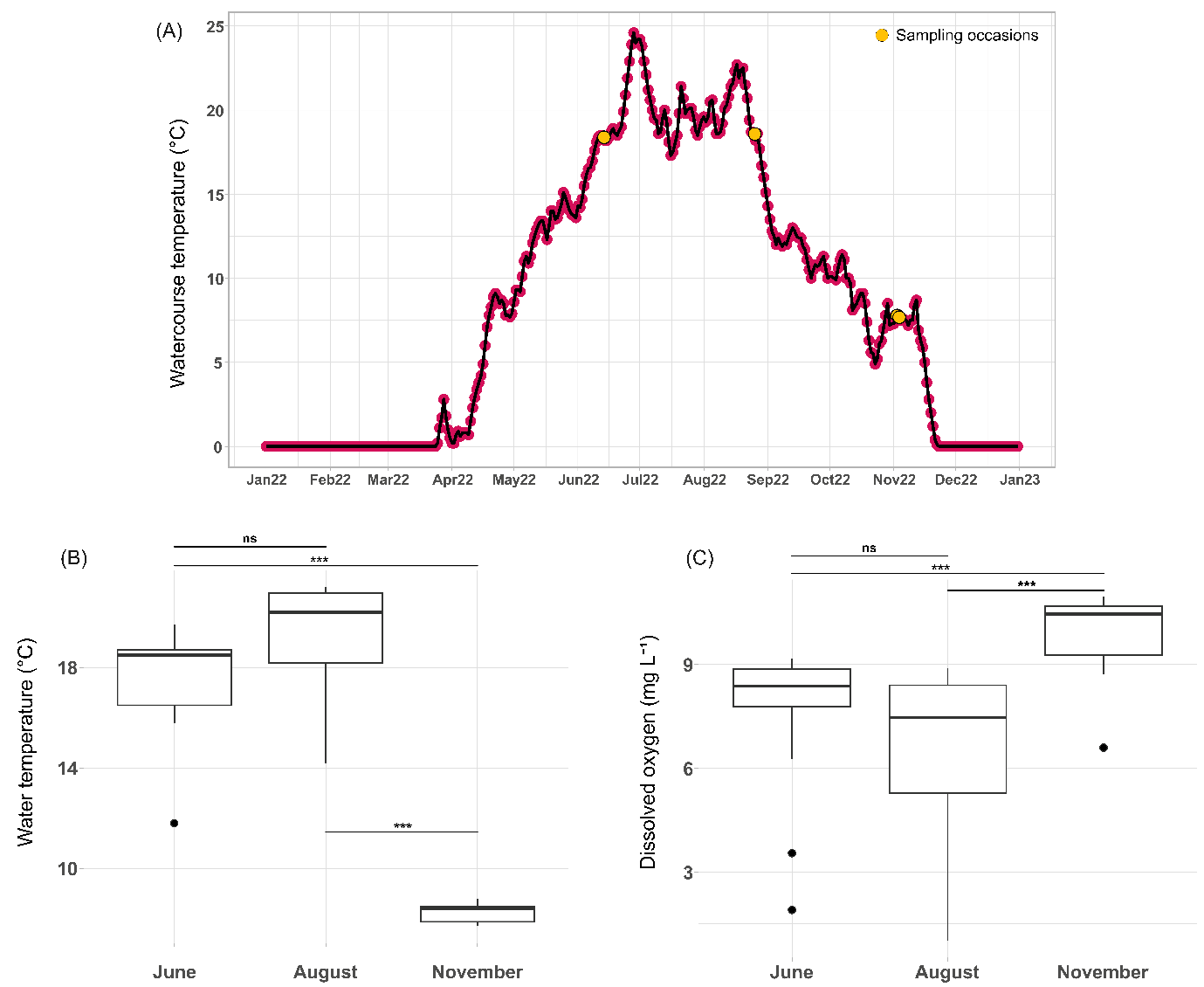


**Figure S2.** (A) Modeled watercourse temperature at the outlet of the system, with the sampling occasions indicated. (B) *In situ* water temperature measurements and (C) dissolved oxygen concentrations across all sampled locations, grouped by month. Statistical comparisons (B, C) were performed with Kruskal–Wallis tests (see main text), followed by Dunn’s *post hoc* pairwise comparisons. Significance levels: *** *p* < 0.001; ns = not significant.

*Supplementary results: Stratification status of lakes*


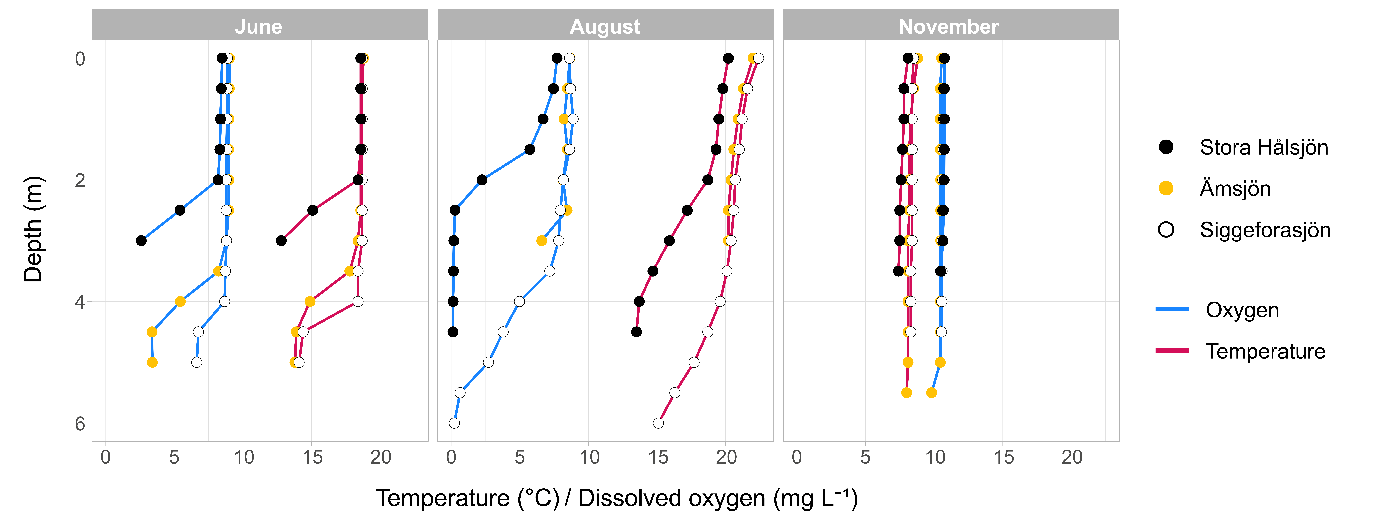


**Figure S3.** Water temperature and dissolved oxygen concentration profiles in three lakes within the catchment, used to assess stratification status across seasons. Complete mixing of the lakes was observed only during the November sampling campaign.

*Supplementary results: Total carbon, nitrogen and phosphorus*


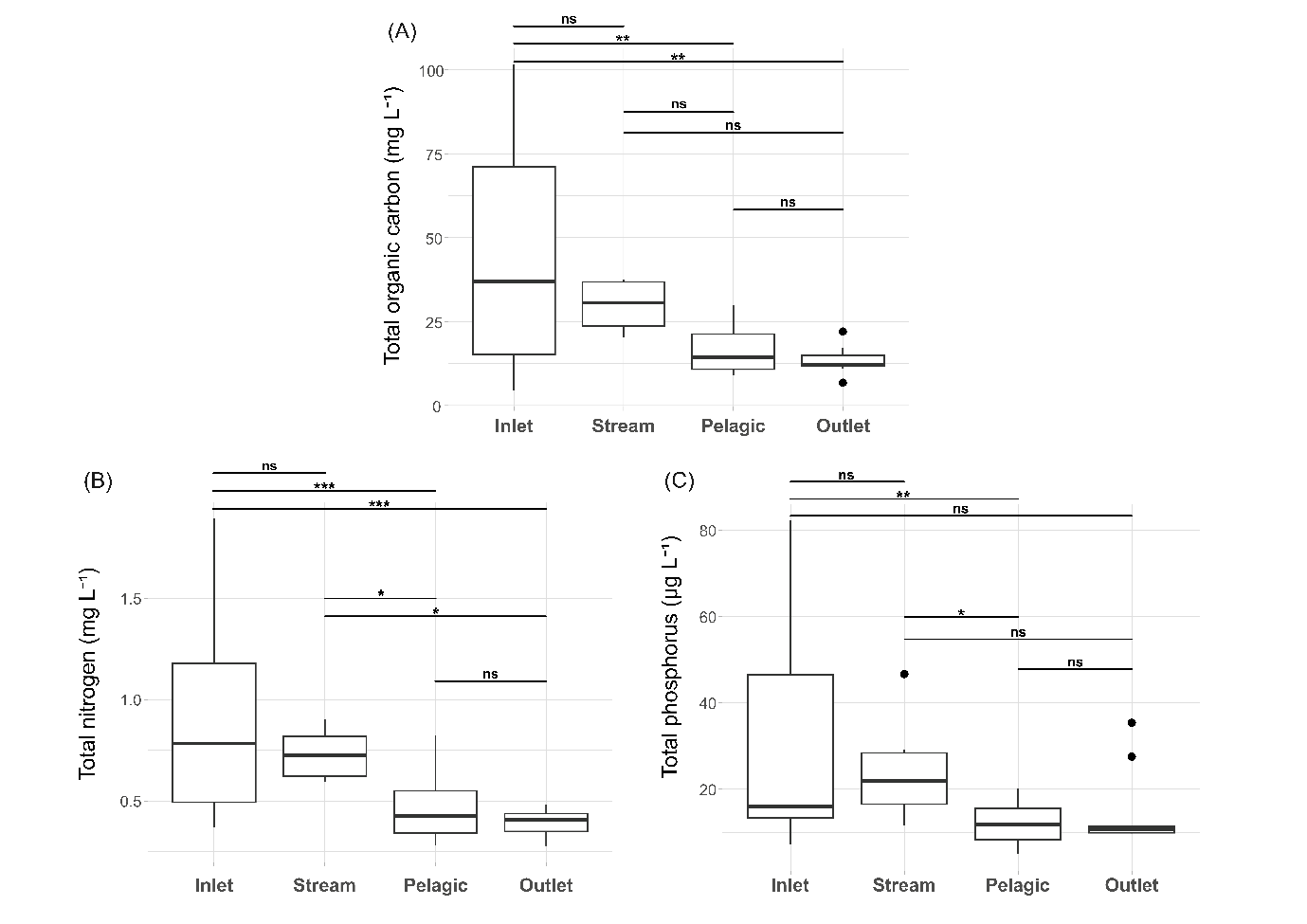


**Figure S4.** (A) Total organic carbon, (B) total nitrogen and (C) total phosphorus concentrations across habitat types during the three sampling campaigns. Statistical analyses were conducted using two-way ANOVAs (see Table S6), followed by *post hoc* comparisons with Tukey’s HSD test. Significance levels: *** *p* < 0.001; ** *p* < 0.01; * *p* < 0.05; ns = not significant.

**Table S6.** Results of two-way ANOVAs (Type III sums of squares) testing the effects of habitat type, sampling month and their interaction on the concentrations of total organic carbon, total nitrogen and total phosphorus. All variables were naturally log-transformed prior to analysis to meet test assumptions. Significant *p* values (*p* < 0.05) are shown in bold.

| **Variable** (naturally log-transformed) | **Effect** | **df** | **F value** | ***p* value** |
| --- | --- | --- | --- | --- |
| Total Organic Carbon | Habitat type | 3, 41 | 4.92 | **0.005** |
|  | Sampling month | 2, 41 | 2.08 | 0.137 |
|  | Interaction | 6, 41 | 0.63 | 0.707 |
| Total Nitrogen | Habitat type | 3, 40 | 2.90 | **0.047** |
|  | Sampling month | 2, 40 | 0.49 | 0.619 |
|  | Interaction | 6, 40 | 0.38 | 0.889 |
| Total Phosphorus | Habitat type | 3, 39 | 6.57 | **0.001** |
|  | Sampling month | 2, 39 | 1.95 | 0.155 |
|  | Interaction | 6, 39 | 0.87 | 0.522 |

*Supplementary results: Bacterial abundance*


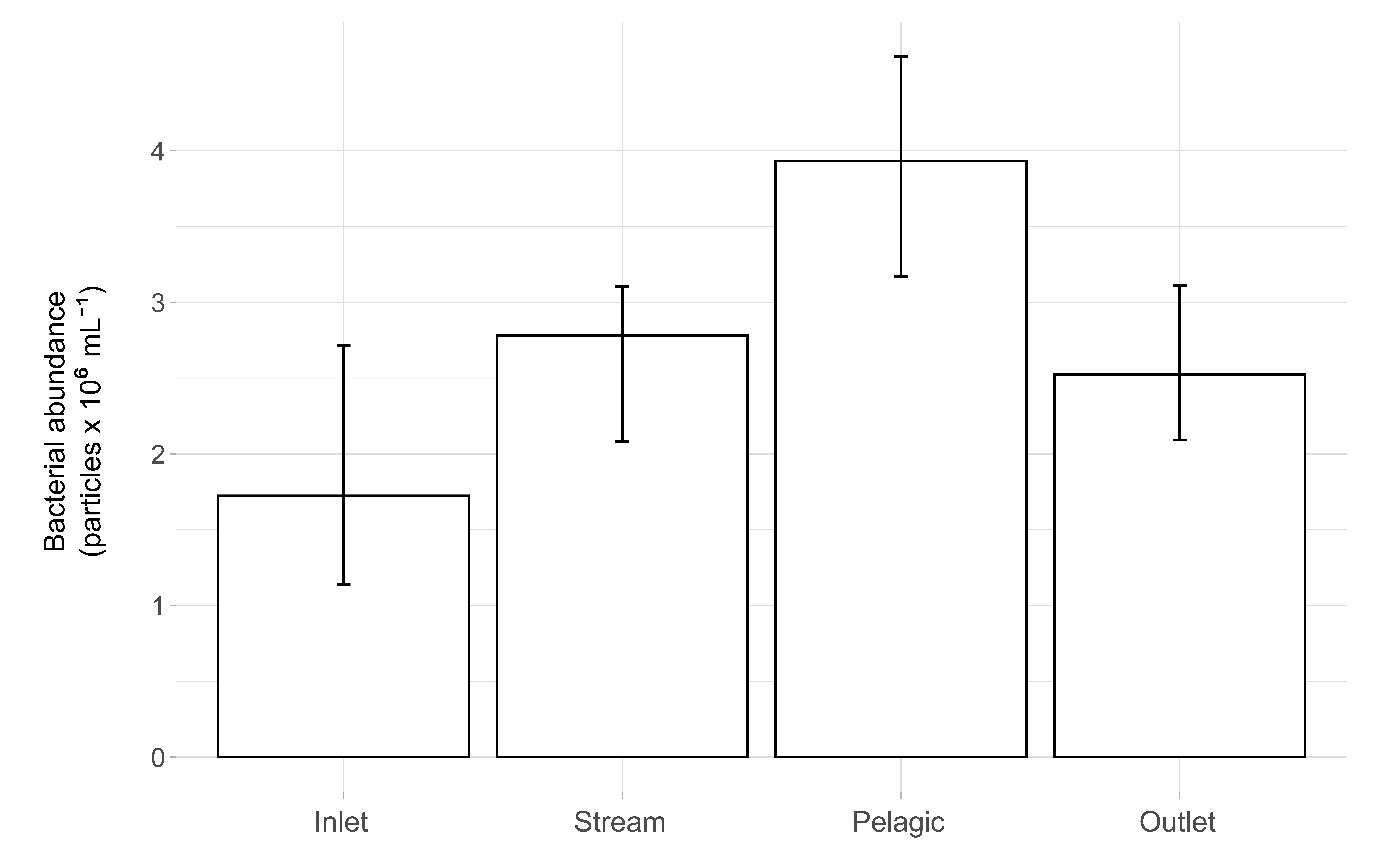


**Figure S5.** Bacterial abundance across habitat types during the three sampling months. Bars represent median values for each group; error bars show 50% confidence intervals around the median.


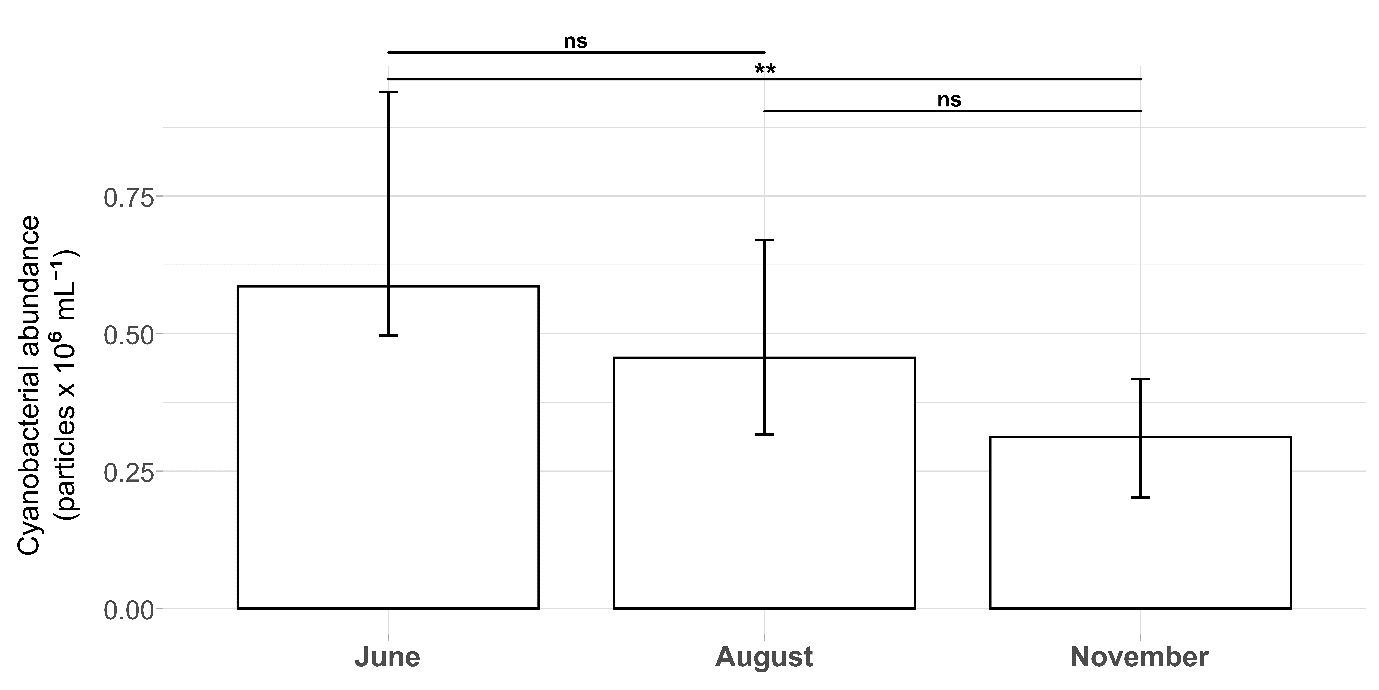


**Figure S6.** Cyanobacterial abundance across sampling months. Bars represent median values for each group; error bars show 50% confidence intervals around the median. Statistical differences were assessed using a Kruskal–Wallis test (see main text), followed by Dunn’s *post hoc* pairwise comparisons. Significance levels: ** *p* < 0.01; ns = not significant.

**Table S7.** List of samples from the three sampling campaigns that were **not** successfully sequenced for one of the nucleic acid fractions (DNA/RNA) of the 18S rRNA gene. Sampling months are abbreviated as J: June, A: August and N: November. The names of the biological samples are further explained in Table S1.

| **Sample** | **Month** | **Fraction** |
| --- | --- | --- |
| STH-Inlet1 | N | DNA |
| STH-Inlet2 | J | DNA |
| STH-Inlet2 | N | DNA |
| STH-Pelagic1 | J | DNA |
| STH-Pelagic2 | J | DNA |
| HAL-2 | J | DNA |
| HAL-2 | N | RNA |
| SIG-Inlet3 | N | DNA |

*Supplementary results: Rarefaction curves*


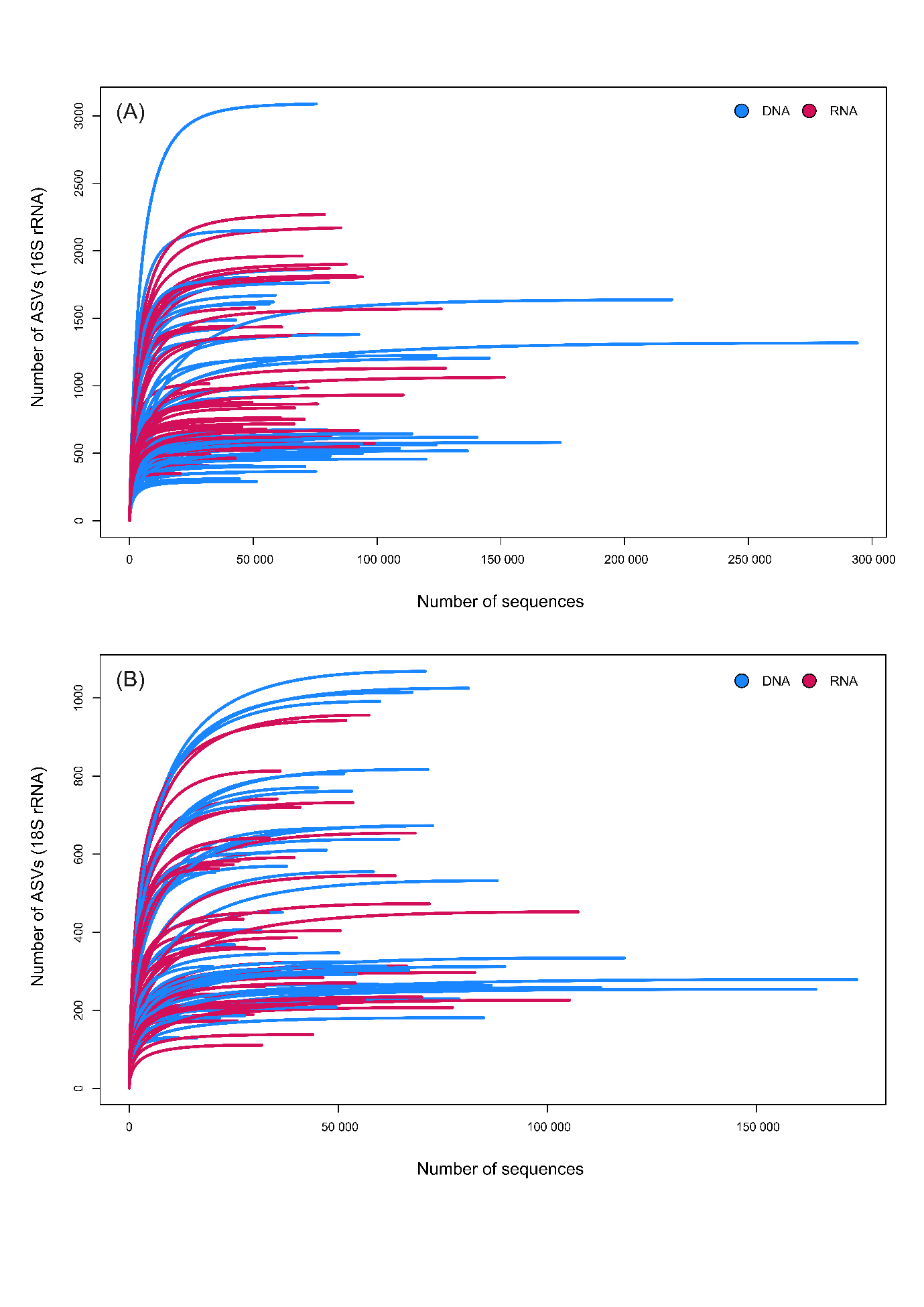


**Figure S7.** Rarefaction curves for (A) 16S rRNA and (B) 18S rRNA datasets. Curves represent sequencing depth across samples, with DNA (genes) and RNA (transcripts) fractions distinguished by color.

*Supplementary results: Rank abundance curves*


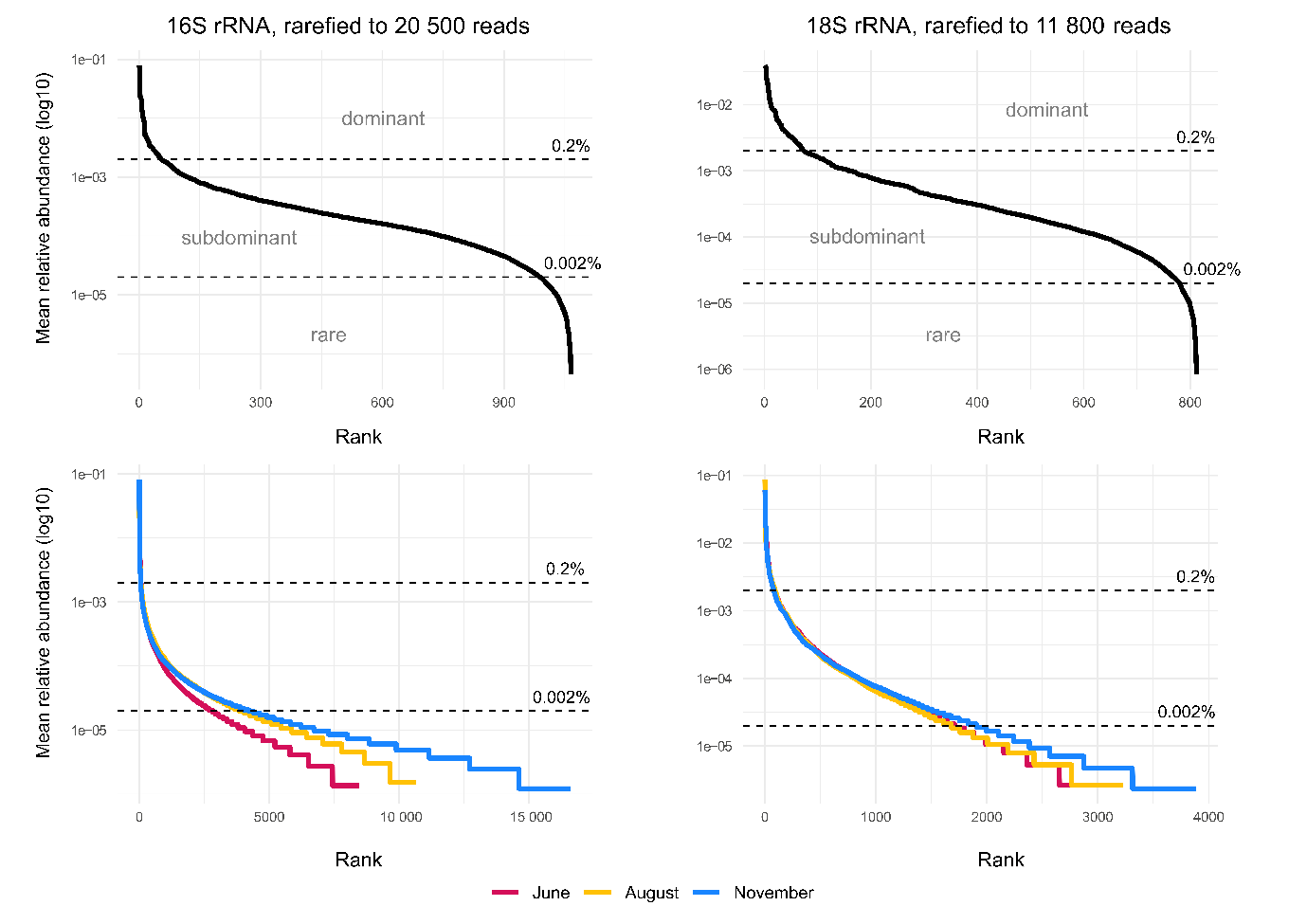


**Figure S8.** Rank abundance plots for the 16S rRNA (left panels) and 18S rRNA datasets (right panels). In the upper panels, amplicon sequence variants (ASVs) with identical mean relative abundances were assigned the same rank. In the lower panels, each ASV was assigned a unique rank. Based on these plots, ASVs were classified into three abundance groups: dominant (relative abundance ≥ 0.2%), subdominant (> 0.002% and < 0.2%) and rare (< 0.002%).

*Supplementary results: Occupancy plots*


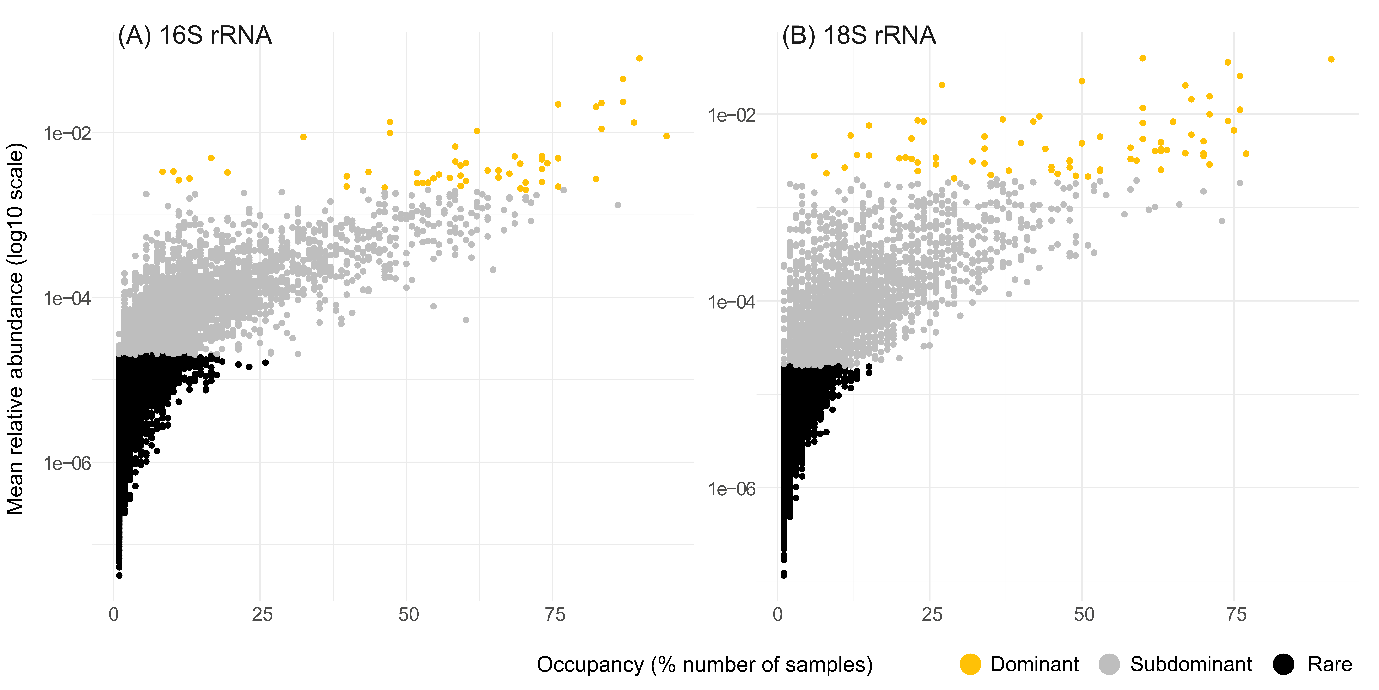


**Figure S9.** Occupancy of amplicon sequence variants (ASVs) from non-rarefied datasets. (A) 16S rRNA (28 126 ASVs) and (B) 18S rRNA (6702 ASVs). ASVs are colored by their abundance groups: dominant (relative abundance ≥ 0.2%), subdominant (> 0.002% and < 0.2%) and rare (< 0.002%).

*Supplementary results: Beta diversity for DNA and RNA communities*


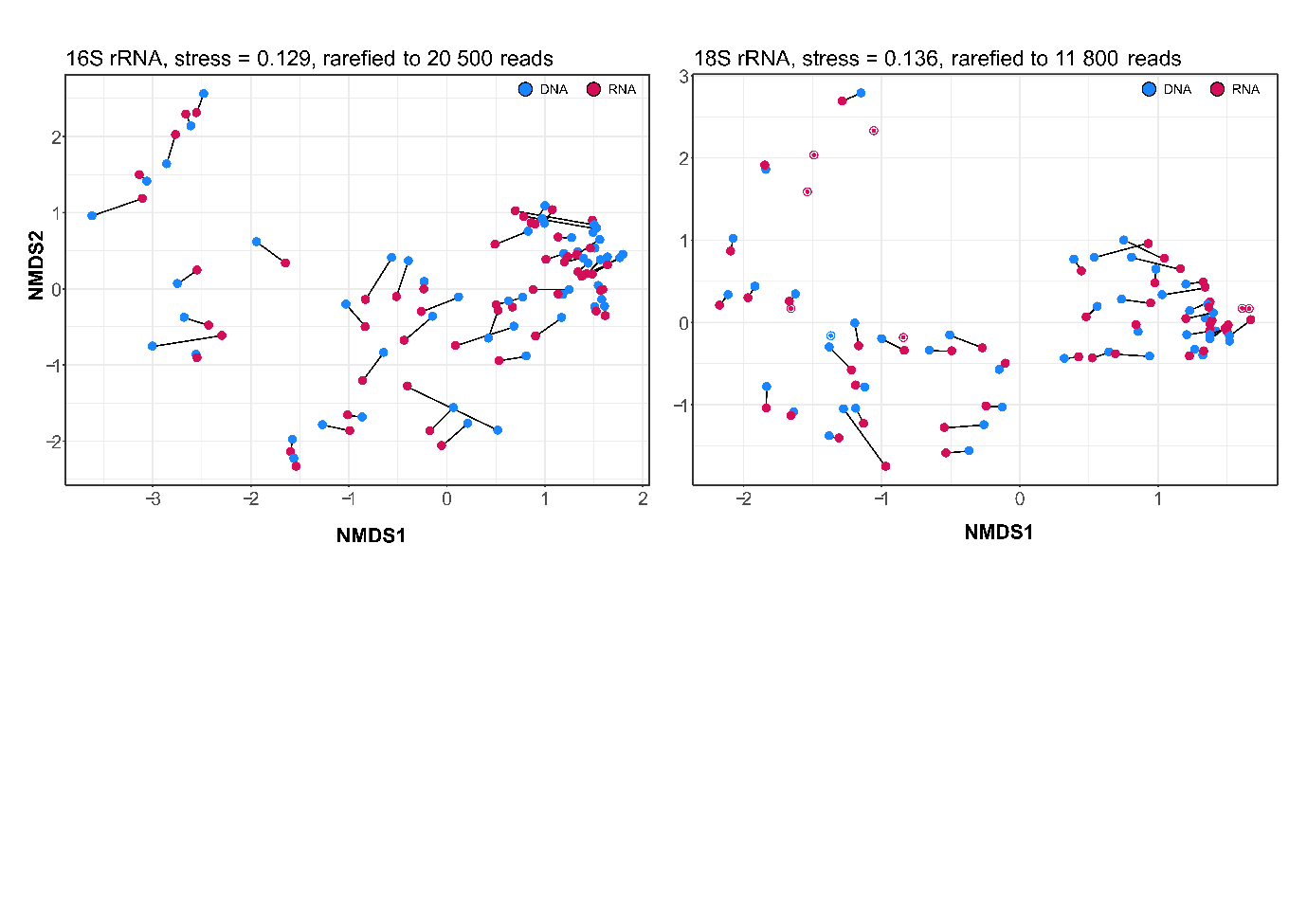


**Figure S10.** Non-metric multidimensional scaling (NMDS) plots (amplicon sequence variant level), calculated using Bray-Curtis dissimilarity matrices for bacterial (left) and microeukaryotic (right) communities in all locations across the three sampling campaigns. Symbols represent individual samples (108 for 16S rRNA and 100 for the 18S rRNA dataset) and they are colored according to the type of nucleic acid fraction sequenced (DNA or RNA). DNA and RNA samples that are connected with a line belong to the same biological sample. In the 18S rRNA dataset, there were eight samples for which the DNA or RNA pair was neither successfully amplified nor sequenced (see Table S7). Therefore, they have no connecting line and appear opaque. NMDS stress values and rarefaction depths are also indicated on the plots.

**Table S8.** Analysis of Bray-Curtis dissimilarities among all bacterial communities (rarefied to 20 500 reads). Differences in dispersion were assessed using the *betadisper* function, followed by ANOVA tests. PERMANOVAs were conducted using the *adonis2* function (both from vegan in R). For PERMANOVAs including interaction terms, the results for both main effects and the interaction term are reported. The stress value for the NMDS plot is also provided. "Fraction" refers to the nucleic acid fraction sequenced (rRNA genes or transcripts), "month" to the sampling month and "type" to the habitat type. Statistically significant *p* values are shown in bold.

| **Group** | **Number of ASVs** | **Factor** | df | **Dispersion** (*betadisper*) | | df | **PERMANOVA** (*adonis2*) | | | **NMDS stress** |
| --- | --- | --- | --- | --- | --- | --- | --- | --- | --- | --- |
|  |  |  |  | F value | *p* value |  | F value | R^2^ | *p* value |  |
| All 16S rRNA communities | 25 957 | fraction | 1,106 | 2.28 | 0.134 | 1,106 | 5.24 | 0.047 | **<0.001** | 0.129 |
|  |  | month | 2,105 | 0.81 | 0.450 | 2,96 | 4.24 | 0.057 | **<0.001** |  |
|  |  | type | 3,104 | 67.34 | **<0.001** | 3,96 | 10.48 | 0.211 | **<0.001** |  |
|  |  | month-type interaction |  | | | 6,96 | 2.16 | 0.087 | **<0.001** |  |

**Table S9.** Analysis of Bray-Curtis dissimilarities among all microeukaryotic communities (rarefied to 11 800 reads). Differences in dispersion were assessed using the *betadisper* function, followed by ANOVA tests. PERMANOVAs were conducted with *adonis2*. For PERMANOVAs including interaction terms, the results for both main effects and the interaction term are reported. The stress value for the NMDS plot is also provided. "Fraction" refers to the nucleic acid fraction sequenced (rRNA genes or transcripts), "month" to the sampling month and "type" to the habitat type. Statistically significant *p* values are shown in bold.

| **Group** | **Number of ASVs** | **Factor** | df | **Dispersion** (*betadisper*) | | df | **PERMANOVA** (*adonis2*) | | | **NMDS stress** |
| --- | --- | --- | --- | --- | --- | --- | --- | --- | --- | --- |
|  |  |  |  | F value | *p* value |  | F value | R^2^ | *p* value |  |
| All 18S rRNA communities | 5926 | fraction | 1,98 | 0.004 | 0.951 | 1,98 | 3.51 | 0.035 | **<0.001** | 0.136 |
|  |  | month | 2,97 | 0.46 | 0.634 | 2,88 | 5.89 | 0.087 | **<0.001** |  |
|  |  | type | 3,96 | 15.47 | **<0.001** | 3,88 | 6.44 | 0.143 | **<0.001** |  |
|  |  | month-type interaction |  | | | 6,88 | 2.61 | 0.116 | **<0.001** |  |

*Supplementary results: Cytometric diversity*

**Table S10.** Analysis of Bray-Curtis dissimilarities among all cytometric fingerprints. Differences in dispersion were assessed with *betadisper*, followed by ANOVA tests. PERMANOVAs were conducted using *adonis2*. The stress value for the NMDS plot is also provided. "Month" refers to the sampling month and "type" to the habitat type. Statistically significant *p* values are shown in bold.

| **Group** | **Number of cells** | **Factor** | df | **Dispersion** (*betadisper*) | | **PERMANOVA** (*adonis2*) | | | **NMDS stress** |
| --- | --- | --- | --- | --- | --- | --- | --- | --- | --- |
|  |  |  |  | F value | *p* value | F value | R^2^ | *p* value |  |
| All communities | 7000 | month | 2,51 | 0.66 | 0.520 | 2.53 | 0.090 | **0.013** | 0.108 |
|  |  | type | 3,50 | 2.57 | 0.065 | 5.23 | 0.239 | **<0.001** |  |
|  |  | month-type interaction | 11,42 |  | | 3.36 | 0.468 | **<0.001** |  |


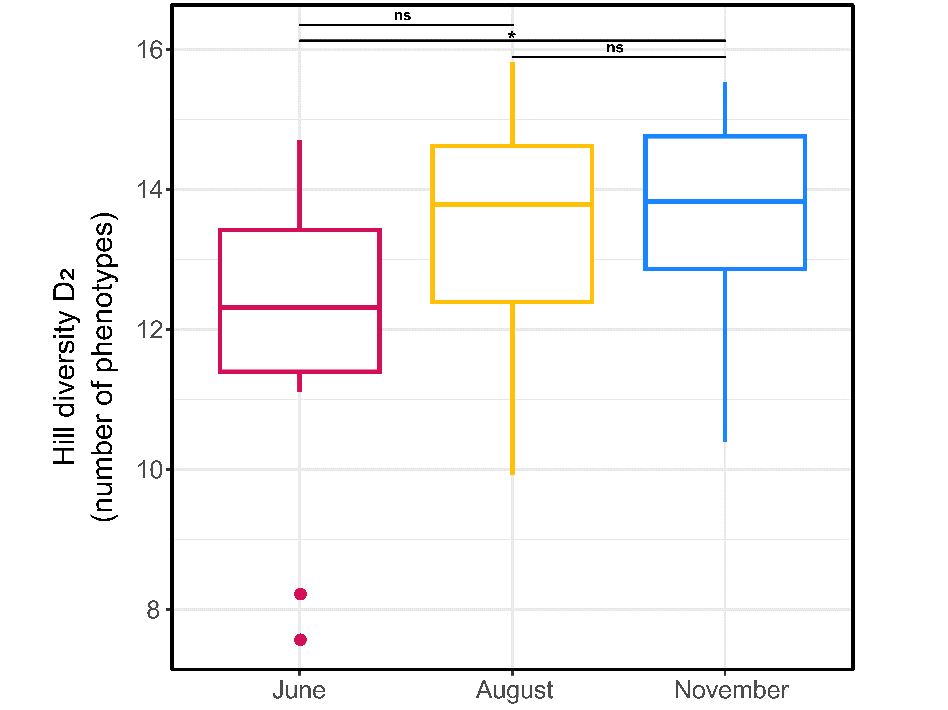


**Figure S11.** Phenotypic diversity, expressed as Hill diversity D_2_, based on cytometric fingerprints of bacterial particles. Boxplots are colored by sampling month. Statistical results are based on a Kruskal–Wallis test (see main text) followed by Dunn’s *post hoc* comparisons. Significance levels: * *p* < 0.05; ns = not significant.

**
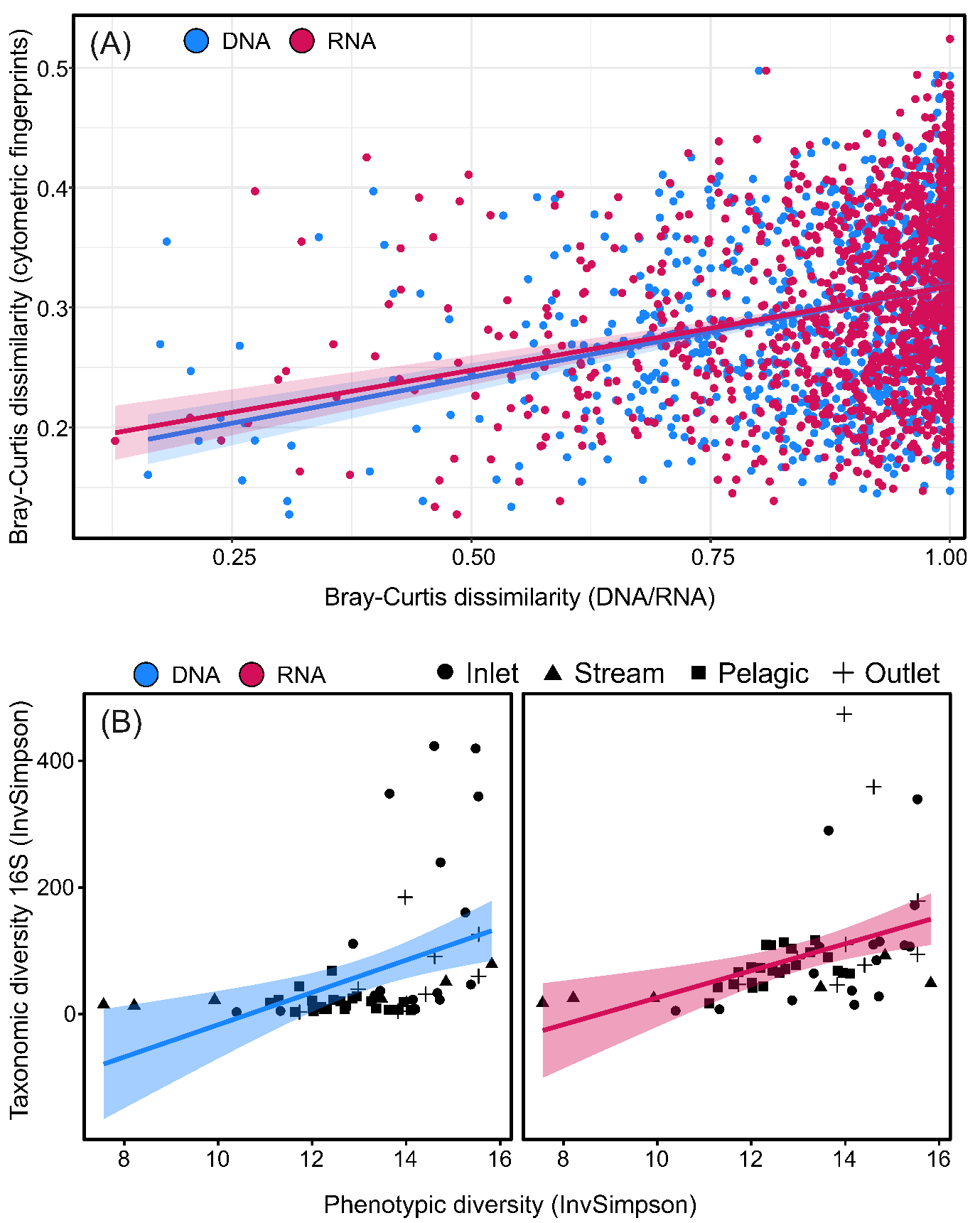
**

**Figure S12.** (A) Relationship between Bray-Curtis dissimilarities derived from cytometric fingerprints and 16S rRNA-based taxonomic profiles for the phylum Cyanobacteria. Each point represents a pairwise comparison between samples, with colors indicating the nucleic acid fraction sequenced ("DNA" for 16S rRNA genes and "RNA" for 16S rRNA transcripts). The 16S rRNA datasets were rarefied to 20 500 reads per sample, yielding 333 cyanobacterial taxa in total, 219 amplicon sequence variants (ASVs) in the DNA and 270 ASVs in the RNA dataset. (B) Correlation between Inverse Simpson diversity indices calculated from 16S rRNA-based taxonomic profiles (total DNA and RNA communities) and phenotypic diversity estimated via flow cytometry. Each panel corresponds to a different nucleic acid fraction, with point shapes denoting habitat type.

*Supplementary results: Phantom taxa*

**Table S11.** Summary of the proportion of phantom taxa across biological samples for the 16S and 18S rRNA datasets. Values are reported as medians and interquartile ranges (IQR) for both ASV-level data and OTUs clustered at 97% sequence similarity.

| **rRNA gene** |  | **Median (%)** | **IQR (%)** |
| --- | --- | --- | --- |
| 16S | **ASVs** | 34.43 | 24.11 - 43.72 |
| 18S |  | 23.87 | 15.79 - 29.86 |
| 16S | **OTUs** | 31.85 | 22.14 - 40.92 |
| 18S |  | 21.60 | 14.48 - 27.06 |

*Supplementary results: Spatial patterns of microbial reactivity*

**
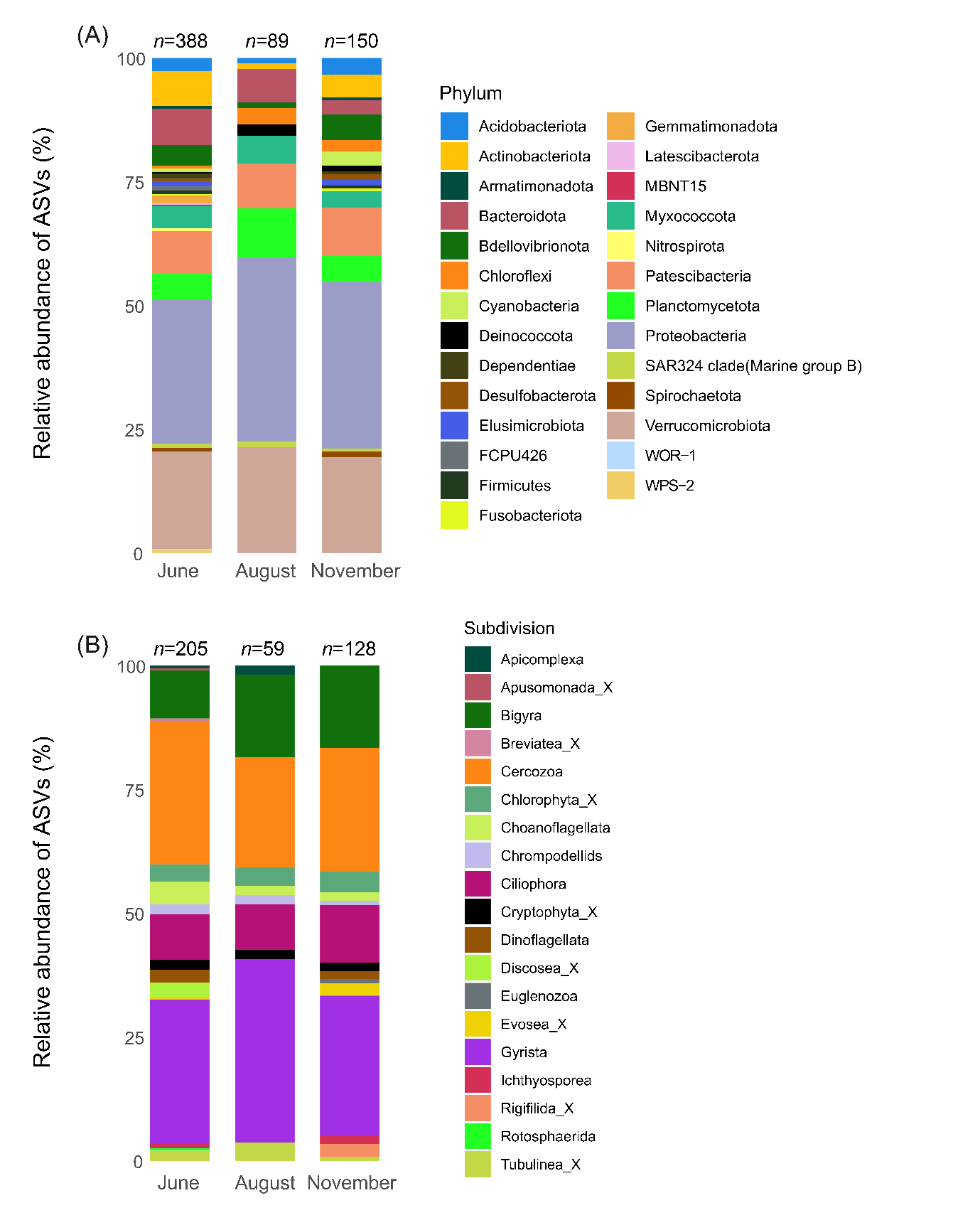
**

**Figure S13.** Taxonomic composition of amplicon sequence variants (ASVs) shared across all lake transects (see Table S5), grouped by sampling month. (A) 16S rRNA: Relative abundance of bacterial phyla. (B) 18S rRNA: Relative abundance of microeukaryotic subdivisions. Sample size (*n*) is indicated for each bar.

**Table S12.** Number of amplicon sequence variants (ASVs) shared across all four locations within each transect, grouped by lake system, sampling month and rRNA gene. Additional details about the selected lake transects are provided in Table S5. System abbreviations: AMS: lake Ämsjön, STH: lake Stora Hålsjön, HAL: Hålsjöbäcken ("connecting stream"), TAR: lake Tarmlången, SIG: lake Siggeforasjön. Sampling months are abbreviated as J: June, A: August and N: November. A dash (–) indicates that no transect was available for the corresponding combination of sampling month and lake system in the 18S rRNA dataset due to unsuccessful sequencing of either the DNA or RNA fraction (see Table S7).

| **Transect** | **System** | **Month** | **Number of shared ASVs** | |
| --- | --- | --- | --- | --- |
|  | | | 16S rRNA | 18S rRNA |
| 1 | AMS | J | 250 | 118 |
| 1 | AMS | A | 14 | 8 |
| 1 | AMS | N | 6 | 12 |
| 2 | STH | J | 13 | – |
| 2 | STH | A | 5 | 1 |
| 2 | STH | N | 20 | – |
| 3 | TAR | N | 29 | 59 |
| 4 | SIG | J | 125 | 87 |
| 4 | SIG | A | 70 | 50 |
| 4 | SIG | N | 124 | 57 |

*Supplementary results: Community assembly*

**Table S13.** Results of Wilcoxon rank-sum tests comparing the relative contributions of dispersal and selection processes to community assembly in bacterial (16S rRNA) and microeukaryotic (18S rRNA) communities, based on DNA (rRNA genes) and RNA (rRNA transcripts) fractions. Each test compares the distributions of the inferred ecological process importance across within-habitat pairwise community turnovers (non-pelagic and pelagic sites). Dispersal includes the combined effects of homogenizing dispersal and dispersal limitation, while selection encompasses the combined effects of homogeneous and heterogeneous selection, as inferred by the iCAMP framework. Statistically significant *p* values are shown in bold.

| **Ecological process** | **rRNA gene** | **Fraction** | **Pairwise turnovers** | **W statistic** | ***p* value** |
| --- | --- | --- | --- | --- | --- |
| Dispersal | 16S | DNA | 128 | 3802 | **<0.001** |
|  |  | RNA | 128 | 3614 | **<0.001** |
|  | 18S | DNA | 96 | 1492 | **<0.001** |
|  |  | RNA | 127 | 3450 | **<0.001** |
| Selection | 16S | DNA | 128 | 405 | **<0.001** |
|  |  | RNA | 128 | 362 | **<0.001** |
|  | 18S | DNA | 96 | 960 | 0.816 |
|  |  | RNA | 127 | 1272 | **0.001** |

**References**

Menzel DW, Corwin N. The Measurement of Total Phosphorus in Seawater Based on the Liberation of Organically Bound Fractions by Persulfate Oxidation1. *Limnology and Oceanography* 1965;**10**:280–2.

Murphy J, Riley JP. A Single-Solution Method for the Determination of Soluble Phosphate in Sea Water. *J Mar Biol Ass* 1958;**37**:9–14.

Ning D, Yuan M, Wu L et al. A quantitative framework reveals ecological drivers of grassland microbial community assembly in response to warming. *Nat Commun* 2020;**11**:4717.

Paradis E, Claude J, Strimmer K. APE: Analyses of Phylogenetics and Evolution in R language. *Bioinformatics* 2004;**20**:289–90.

Props R, Monsieurs P, Mysara M et al. Measuring the biodiversity of microbial communities by flow cytometry. *Methods in Ecology and Evolution* 2016;**7**:1376–85.

Schliep KP. phangorn: phylogenetic analysis in R. *Bioinformatics* 2011;**27**:592–3.

Wright E S. Using DECIPHER v2.0 to Analyze Big Biological Sequence Data in R. *The R Journal* 2016;**8**:352.
